# Pseudocapacitive Titanium Oxynitride Nanowires for Ultrahigh Capacitance Supercapacitors

**DOI:** 10.1021/acsanm.5c04882

**Published:** 2026-01-26

**Authors:** Sheilah Cherono, Panupong Jaipan, Zixiao Shi, Simon Gelin, Joan Ejeta, Ikenna Chris-Okoro, Mengxin Liu, Ghanashyam Gyawali, Wisdom Akande, Jonghyun Choi, Swapnil Nalawade, Shobha Mantripragada, Ram K. Gupta, James D. Schall, Kristen L. Rhinehardt, Ismaila Dabo, Shyam Aravamudhan, Bishnu P. Bastakoti, David A. Muller, Dhananjay Kumar

**Affiliations:** † Department of Mechanical Engineering, 3616 North Carolina Agricultural and Technical State University, Greensboro, North Carolina 27411, United States; ‡ Department of Chemistry and Chemical Biology, Cornell University, Ithaca, Newyork 14853, United States; § Department of Materials Science and Engineering, and Wilton E. Scott Institute for Energy Innovation, 6612 Carnegie Mellon University, Pittsburgh, Pennsylvania 15213, United States; ∥ Department of Computational Science and Engineering, North Carolina A&T State University, Greensboro, North Carolina 27411, United States; ⊥ Department of Chemistry, Kansas Polymer Research Centre, 6594 Pittsburgh State University, Pittsburgh, Kansas 66762, United States; # Department of Nanoengineering, 601950 Joint School of Nanoscience and Nanoengineering (JSNN), Greensboro, North Carolina 27401, United States; ¶ Department of Chemistry, North Carolina A&T State University, Greensboro, North Carolina 27411, United States; $ School of Applied and Engineering Physics, 5922 Cornell University, Ithaca, New York 14853, United States

**Keywords:** titanium oxynitride, nanowires, thin films, supercapacitance, pseudocapacitors, charge-transfer, surface orientation, pulsed laser deposition

## Abstract

High-quality, multifunctional two-dimensional (2D) titanium oxynitide (TiNO) thin films and one-dimensional (1D) TiNO nanowires have been synthesized using a pulsed laser deposition, a simple, fast, and congruent evaporation method. First-principles calculations as a function of surface orientation and termination indicate that surface oxidation of TiNO nanowires can stabilize the (110) orientation observed experimentally. The specific capacitance value for the TiNO nanowire samples (2725 mF/cm^2^) has been found to be nearly six times more than that of the TiNO thin film samples (400 mF/cm^2^), which is attributed to the high packing density of TiNO nanowires over a given area. The nanowire samples have also been found to exhibit a significantly higher energy density (1.35 μWh/cm^2^) than the TiNO thin-film samples (0.33 μWh/cm^2^). Thus, the TiNO material system in thin-film and nanowire forms has been demonstrated to be a promising candidate for use as an electrode material in supercapacitors and other charge-storage applications.

## Graphical abstract

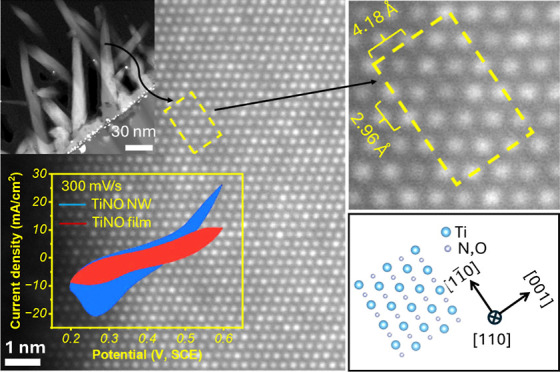

## 1 Introduction

Among alternative energy sources, supercapacitors have been of great interest in electrochemical energy storage devices due to their fast charge–discharge capability, high specific capacitance, long life, safety, and reliability.^1–3^ The development of electrode materials for supercapacitor applications and the demands of integrated and miniaturized multifunctional electronic devices have, therefore, been increasing.^3–5^ Recent efforts toward improving the electrochemical supercapacitors have included synthesizing novel material systems, developing a fundamental understanding of the charge transfer process, optimizing electrolyte composition, and designing robust device architecture. In materials design and synthesis, high-entropy materials have emerged as the most promising candidates for supercapacitor applications due to their compositional complexity, synergistic elemental interactions, and tunable d-band centers, which collectively enhance charge-transfer kinetics, electrical conductivity, and redox activity.^6^ Modulation of the d-band center has been shown to directly influence the adsorption energies of electrolyte ions and intermediates, leading to improved capacitance, rate capability, and cycling stability in supercapacitor electrodes. Complementary to this approach, transition metal oxide nanostructures with intentional dopant incorporation have demonstrated multifunctional electrochemical behaviors. For example, strontium-doped molybdenum oxide nanoparticles exhibit enhanced electrochemical sensing of diclofenac, along with improved energy storage and photocatalytic activity, attributed to defect generation, band-structure modification, and increased active surface sites.^7^

Based on their charge-storage mechanisms, supercapacitors are typically classified as electrostatic or electric double-layer capacitors (EDLCs) and pseudocapacitors.^2^ EDLCs represent a type of supercapacitor storing their charge via the electrostatic charge adsorption at the electrode/electrolyte interface with no faradaic reactions. Carbon-based materials (e.g., carbon nanotube, graphene, and nanoporous carbon) are most attractive (EDLCs) due to their low cost, abundance in nature, high conductivity, and excellent cycling stability. However, carbon-based materials typically exhibit relatively low specific capacitances, low energy densities, and high contact resistance.^8^ In contrast, pseudocapacitors represent a type of supercapacitors that exhibit a charge storage mechanism by a faradaic redox reaction at or near the surface of the active electrode materials. Pseudocapacitive materials, such as RuO_2_, IrO_2_, and MnO_2_, have shown much higher capacitance than carbon-based microdevices.^4,9–12^ The limitations in the use of these transition metal oxide-based pseudosupercapacitor arises either due to their high cost, poor stability, and low conductivity.^4,13–15^ Therefore, there are significant efforts underway to develop economical and pragmatic methods for the synthesis of transition metal-based oxynitrides (TMONs) materials for supercapacitor applications due to their good electrochemical properties, good chemical resistance, and high thermal stability.^16–20^ The TMONs can be regarded as nitride derivatives of transition-metal oxides or oxide derivatives of transition-metal nitrides. Wang et al.^21^ investigated the electrochemical performance of cobalt oxynitride (CoON) electrodes, reporting a specific capacitance of 1246 F/g and 97.8% capacitance retention after 8000 cycles. Ruan et al.^22^ have developed 3D porous micropillars of molybdenum oxynitride (MON), which yield remarkable areal specific capacitances of 736.6 mF/cm^2^ at a scan rate of 10 mV/s. Oxynitride photocatalysts, including tantalum oxynitride (TaON) and lanthanum titanium oxynitride (LaTiO_2_N), have also been extensively investigated for their capacity to facilitate water splitting under visible-light irradiation. TaON presents a harmonious blend of visible-light absorption and stability, and LaTiO_2_N demonstrates advantageous band-edge alignment for comprehensive water splitting.^23^

Titanium oxynitride (TiNO) has recently emerged as a novel material system to be explored in a variety of (photo)­electrochemical^24^ and plasmonic applications.^25,26^ In the present work, we report the feasibility of using a pulsed laser deposition (PLD) method in the fabrication of supercapacitor TiNO 1D nanowires as an alternative route to chemical synthesis methods. In the PLD method, neither hazardous gas nor a binder agent is used to attach the electrode materials to the substrates in contrast with the electrode particle-materials prepared from chemical routes.^27–29^ The TMONs electrodes fabricated using powder chemical precursors suffer from poor adhesion to the current collector lying between binders and electrode materials.^25,30^ Besides, the chemical-route synthesis techniques, including hydrothermal, solvothermal, sol–gel, and surfactant-assisted methods, are time-consuming and complicated, and involve toxic gases in the fabrication reaction.^16^ The advantage of PLD over other physical vapor deposition processes is the fast response, energetic evaporants, and congruent evaporation. Short pulses help maintain high laser power density within a small target area and produce uniform evaporation. In the absence of ions or evaporation sources with hot filaments under vacuum, PLD is possible at ∼100–400 mTorr of reactive gas pressure. Since PLD can transfer the target’s composition to the deposited film, it can be used to fabricate complex alloys whose constituents have vapor pressures that differ by 10^6^. There are relatively few deposition parameters to optimize in PLD, but the range of chemical compositions, structural phases, morphology, microstructure, and film structure attainable is enormous. Due to the embodiment of so many advantageous features and relative ease of operation, PLD has become one of the most widely used techniques for thin film and nanotechnology research, particularly in research institutions.

A substantial improvement in the supercapacitive performance of 1D TiNO nanowires over 2D TiNO thin films has been realized, which is attributed to enhanced effective surface areas for ion diffusion at the TiNO nanowire electrode–electrolyte interface, shown schematically in Figure S1. It should be noted that several advanced transition-metal–based electrodes have demonstrated comparably high specific capacitances reported in the present study. For example, the NC-LDH@NCO/SSM composite electrode delivered an enhanced specific capacitance of 2222.27 F/g, confirming that capacitance values in this range are achievable for hierarchical hybrid architectures.^11^ Similarly, MnCo_2_O_4_ nanospheres have been reported to exhibit a high specific capacity of 2019 F/g at 1 A/g, further supporting the validity of high-performance electrode systems in the literature.^12^

## 2 Experimental Section

A multitarget pulsed laser deposition (PLD) system was used to grow TiNO nanowires and TiNO thin films. In our PLD experiments, a krypton fluoride (KrF) excimer laser (wavelength of 248 nm, pulse duration of 30 ns) was used as a laser source. The depositions were carried out using optimized laser parameters: laser energy density of 5.0 J/cm^2^, laser frequency of 10 Hz, target–substrate distance of 5 cm, substrate temperature of 800 °C, number of laser pulses of 12,000, and (100)-oriented 10 mm × 10 mm × 0.4 mm silicon single-crystal substrates. The details of the PLD optimization process are published elsewhere.^31^ Solid titanium nitride and gold (Au), both with a high purity of 99.99% (Kurt J. Lesker Company) and a diameter of 1 in., were used as target materials for the deposition of TiNO nanowires. A (100)-oriented silicon substrate (10 mm × 5 mm) was used. Before TiNO deposition, the silicon substrate was sequentially cleaned in ethanol, acetone, and methanol for 10 min each. The cleaned silicon substrate was then mounted on the sample heater holder and kept in the vacuum chamber. The chamber was evacuated to a base pressure of 1 × 10^–6^ Torr. The gold film was first deposited on the substrate using 100 laser pulses by ablating the gold target. Subsequently, the Au film was subjected to postdeposition annealing at 200 mTorr N_2_ in the same chamber, without breaking vacuum, at 800 °C for 30 min to generate gold nanodots that serve as catalysts. TiNO nanowires were deposited over the Au nanodots using 12,000 laser pulses at a repetition rate of 10 Hz and laser energy density of ∼5 J/cm^2^ keeping nitrogen pressure (200 mTorr) and substrate temperature (800 °C) the same as during the Au film annealing. The sample was then cooled to room temperature under 400 mTorr of N_2_ and used for various measurements.

The morphologies of TiNO nanowires (lengths, diameters) were studied using a Hitachi SU8000 field-emission scanning electron microscopy (FE-SEM). The atomic structures of TiNO NWs were studied using a high-resolution scanning transmission electron microscope (STEM). The TEM lamella sample preparation was done by cutting the TiNO NWs on the Si substrate along the [100] facet of Si by focused ion beam (FIB) using Thermo Fisher Scientific Helios 5 Dual Beam. Subsequently, the TiNO NWs sample was first coated with a 1 μm-thick carbon and Pt layer to protect the TiNO NWs from ion-beam damage. High-resolution scanning transmission electron microscopy (STEM) and electron energy-loss spectroscopy (EELS) were acquired using a FEI/Thermo Titan Themis CryoS/TEM with a monochromator at 80 kV. The STEM images were taken with a 50 μm condenser aperture and convergence angle of 21.4 mrad to achieve a resolution of 1.2 Å. EELS spectrum was collected with a 50 μm condenser aperture and convergence angle of 21.4 mrad with a 5 times higher dose than STEM imaging with a Gatan Ultrascan detector with energy dispersion of 0.3 eV/channel. The crystallographic characterization was performed using a Bruker D8 advanced X-ray diffractometer (XRD) at 40 kV and 40 mA, and 2θ from 15° to 80° using CuKα radiation. The atomic compositions and oxynitride formation of the samples prepared were analyzed using a Thermo Scientific Escalab Xi + X-ray Photoelectron Spectrometer (XPS) with a spot size of 500 μm and an Al Kαα X-ray source. The water contact angle measurements were performed using a standard sessile drop method, in which a small droplet of deionized water was gently placed onto the surface of the TiNO nanowire and thin-film samples. The contact angle was determined by capturing the droplet profile with an optical camera and analyzing the tangent angle at the solid–liquid–vapor interface using image analysis software.^32^ Multiple measurements at different locations on each sample were typically averaged to ensure reproducibility and minimize surface heterogeneity effects. The contact angles for the TiNO nanowire samples and the TiNO thin-film samples were found to be ∼72° and ∼75°, respectively, suggesting the nanowire samples are slightly more hydrophilic than the thin-film samples. The electrochemical measurements were performed using a VersaStat 4–500 electrochemical workstation (Princeton Applied Research, Oak Ridge, TN, USA) in a conventional three-electrode configuration. One M KOH was used as the electrolyte, with a platinum wire as the counter electrode, a saturated calomel electrode (SCE) as the reference electrode, and TiNO samples as the working electrode, with a working electrode area of 0.2 cm^2^. Cyclic voltammetry (CV) was performed between 0.2 and 0.6 V (vs SCE), and Galvanostatic charge/discharge (GCD) measurements were performed at various current densities. Electrochemical impedance spectroscopy (EIS) was performed during all tests over the frequency range 0.05 Hz to 10 kHz with an ed AC amplitude of 10 mV.

## 3 Results and Discussion

### 3.1 Physics of High-Yield TiNO Nanowire Formation

The formation of Au nanodots that serve as a catalyst during the vapor–liquid–solid process to promote the one-directional growth of TiN/TiNO nanowire was achieved through the annealing of gold films at 800 °C for 30 min. Postdeposition annealing of the Au film was performed in the same deposition chamber at a nitrogen pressure of 200 mTorr; the resulting Au nanodots are shown in Figure 1a. The molten Au nanodots act as catalysts, trapping TiNO vapor molecules from the TiN target during deposition. After the molten Au dots reach supersaturation, the TiNO solid phase precipitates out from the Au droplet catalyst at the liquid–solid interface, forming one-dimensional nanowire structures. The growth mechanism of TiNO nanowires is understood through a physics-based model that begins with the thermodynamic potential and Gibbs free energy. The temporal evolution of the Au catalytic droplet shape and TiN nanowire growth is illustrated in Figure 1b. Following the well-known work of Schmidt et al.^33^ and the Neumann triangle relation,^34^ the relationship between the contact angle of the droplet (β) and the inclination angle (α) of the nanowire side is expressed in terms of different interfacial energies as

1 $$\gamma_{2a}\cos\left(\beta_{0}\right)=\gamma_{0}\cos\left(\alpha \right)-\gamma_{3a}$$

where γ_0_, γ_2α_, and γ_3α_ are the interfacial energies between nitrogen vapor and solid silicon substrate, liquid Au droplet and nitrogen vapor, and liquid Au droplet and solid silicon substrate, respectively. Equation 1 describes the situation before nanowire growth (i.e., at α = 0). This equation is useful in studying the nucleation and growth process of Au islands from a continuous Au film. Then, the TiN begins precipitation, and the formation of the nanowire after supersaturation takes place in the Au droplet with TiN vapor molecules; hence, eq 1 transforms to eq 2 as

2 $$\gamma_{2}\cos\left(\beta \right)=\gamma_{1}\cos\left(\alpha \right)-\gamma_{3}$$

where γ_1_, γ_2_, and γ_3_ are the interfacial energies between TiN vapor and solid TiN nanowire, TiN vapor and Au liquid droplet, and solid TiN nanowire and liquid Au droplet, respectively. Applying the force balance model at the triple-phase line of the droplet (rightmost schematic in Figure 1b), and due to α = 90° at the equilibrium condition, eq 2 becomes γ_2_cos­(β) = -γ_3_. Therefore, an inequality relationship is obtained for the stable growth of nanowires as

$$\gamma_{2}\sin\left(\beta \right)+\gamma_{3}>\gamma_{1}or\;\gamma_{2}>\frac{\gamma_{1}}{\left(\sin\beta -\cos\beta \right)}$$ 3

**Figure 1:**
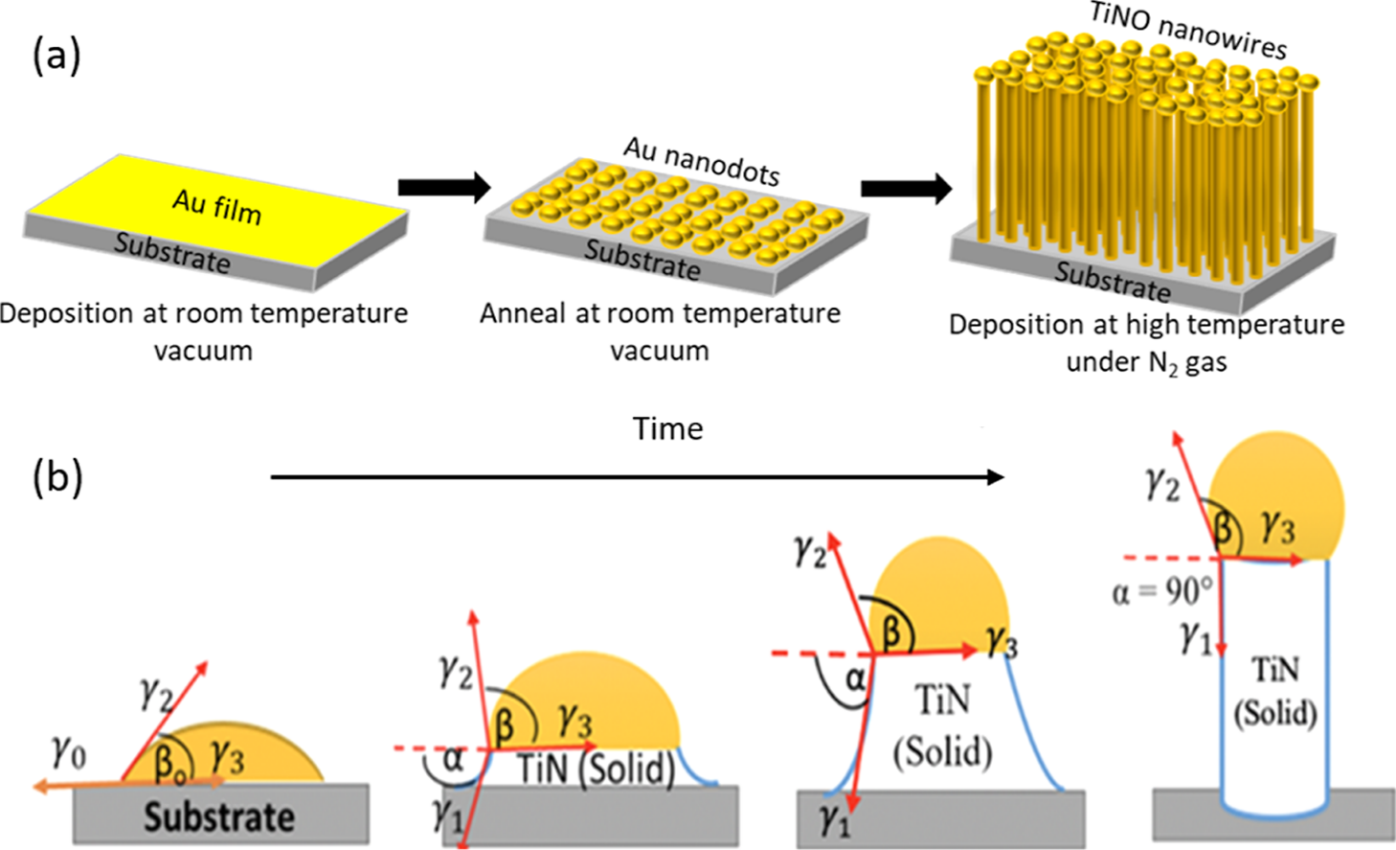
An illustration of (a) TiNO nanowires fabrication process and (b) the shape evolution of the Au catalytic droplet and TiNO/TiN nanowire growth with time.^33^

Since sinβ-cosβ is always smaller than $\sqrt{2}$ , stable growth of the nanowire is realized when $\gamma_{2}>\frac{\gamma_{1}}{\left(\sin\beta -\cos\beta \right)}$ . Using this surface energy criterion, we can determine the threshold for γ_2_ below which the droplet should not be dewretted from the side of the nanowire. Otherwise, the nanowire growth becomes unstable. With the obtained diameter size of gold nanoclusters/catalysts (∼10 nm) and the fabrication temperature at 800 °C, the threshold for γ_2_ ^35^ is estimated to be ∼0.6 J/m^2^, which is consistent with the reports.^33,36,37^ Furthermore, the optimally sized gold nanodots (Figure 2a), uniformly distributed over the substrate, led to the formation of a high-density population of TiNO nanowires on a silicon (100) substrate, as shown in Figure 2b–e. The nanowires are, on average, ∼420 ± 30 nm long and ∼21 ± 2 nm in diameter. Figure 2f displays the surface morphology of the TiNO thin film that was deposited under the same conditions as TiNO nanowires, but without Au catalysts. The TiNO films were found to have a thickness of ∼70 nm. In our study, one key parameter governing the growth of TiNO nanowires is the optimized size of gold nanodots. Therefore, we define the yield of TiNO nanowires as the ratio of the number of catalytic gold nanodots of optimized size per unit area to the number of TiNO nanowires formed per unit area. The yield measured using this approach turns out to be over 80%. The high yield of the PLD process is also reflected in the small time the actual deposition process takes for the formation of nanowires (15000 laser pulses, 10 Hz ≡ 25 min). The approach for determining the number of gold nanodots and TiNO nanowires using FE-SEM is described in the Supporting Information.

**Figure 2:**
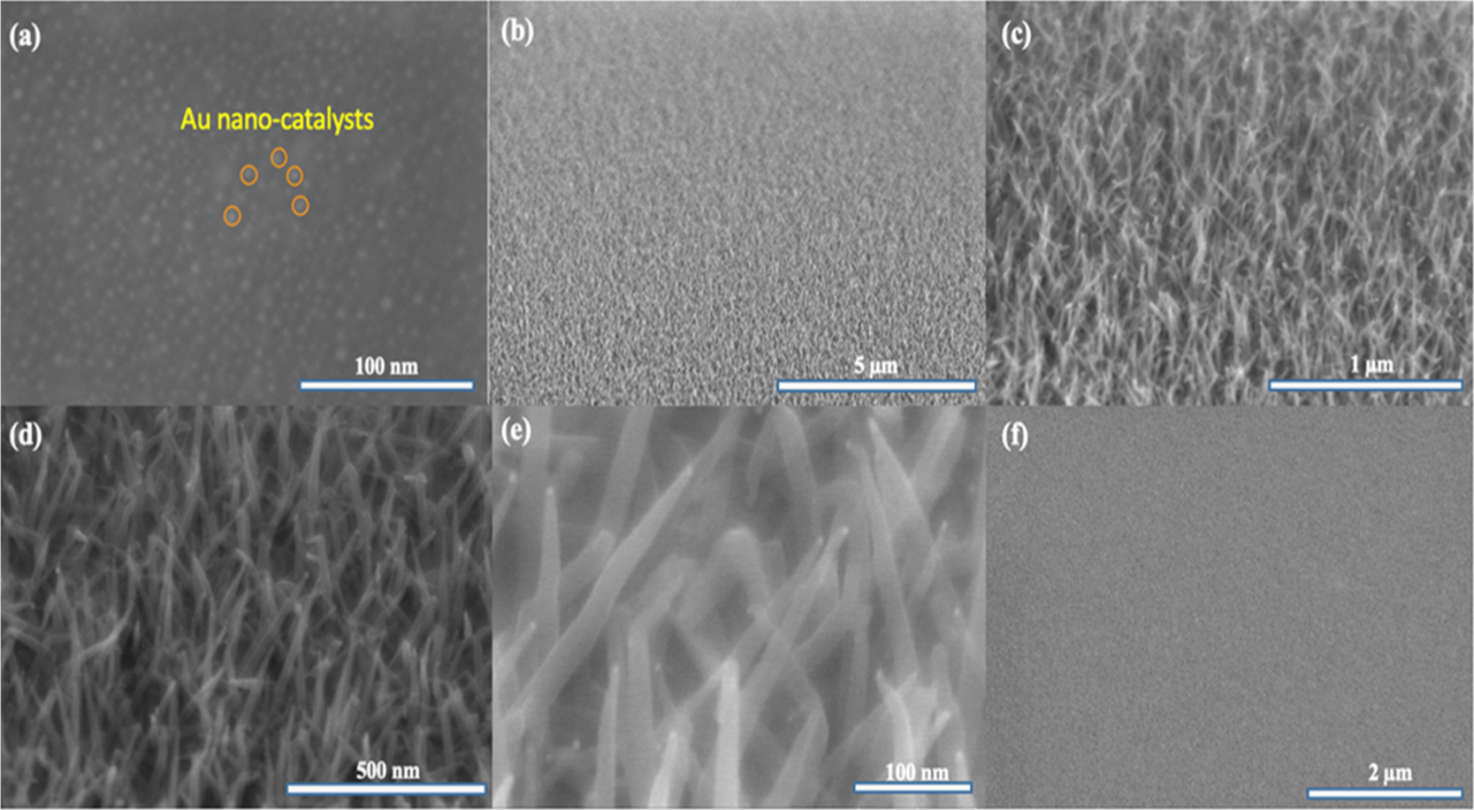
FE-SEM images displaying (a) surface morphology of gold nanoparticle catalysts on the silicon (100) substrate after postannealing with the magnification of 450,000×; FE-SEM images showing the high-dense population of TiNO nanowires with the magnification of 10,000× (b), 50,000× (c), 100,000× (d), and 300,000× (e), respectively; and (f) the surface morphology of the TiNO film grown on silicon (100) with the same conditions as TiNO nanowires, except no Au catalyst deposition for the TiNO film sample.

### 3.2 Experimental and Theoretical Studies of TiNO Phase, Orientation, and Composition

#### 3.2.1 Phase and Orientational Studies Using XRD

X-ray diffraction patterns shown in Figure S2 confirm the presence of TiN and TiO_2_ phases in both samples (TiNO nanowires and TiNO thin film). According to these patterns, the TiNO thin film and nanowire samples consist of a rock-salt face-centered cubic structure of TiNO with (220) orientation (JCPDS card: No. 38-1420) and a rutile tetragonal phase of TiO_2_ peak with (221) orientation (JCPDS card: No. 21-1276). A slight positive shift in the 2θ angles of the (220) plane relative to the TiN phase is taken as evidence for partial oxidation of TiN to the TiNO phase. The presence of TiN and TiO_2_ in the TiNO materials is also consistent with the earlier observation of titanium oxynitride formation in the hollow titanium oxynitride array.^38^ As observed in XRD patterns, the XRD peaks of TiNO nanowires show distinctive peaks of TiN (220) and TiO_2_ (221) planes with significantly higher intensities than those observed in the TiNO film samples. This is attributed to a more crystalline structure in the nanowire samples than in the thin-film samples. More importantly, these XRD results confirm that these nanostructures (nanowires and thin-film) consist of both oxygen and nitrogen species in the samples (TiN_*x*_O_*y*_); there is no single-phase, either TiN or TiO_2_, present alone in the samples. Oxygen diffusion into TiN nanostructures at elevated temperatures is also well documented in other reports 38–40, e.g., in TiN films prepared by arc plasma deposition at high temperatures.^39^ At elevated temperatures, nitrogen from titanium nitride diffuses out, promoting oxygen in-diffusion and, hence, the oxidation of TiN to TiNO.^39,40^ The activation energy of the TiNO formation is relatively small (∼110 kJ/mol)^39^ compared to the activation energy of the TiN formation (202 kJ/mol).^41^ Thus, the likelihood of the formation of a partially oxidized TiN compound is greater than that of pure TiN structures. Furthermore, the rock-salt TiNO oxide component that forms at high temperatures (above 700 °C) can exhibit a nondefective interface with isostructural TiN and form a stable, ordered oxide structure, which helps prevent further oxidation within the structure due to the high energy required to adsorb additional oxygen.^42,43^ In contrast, there are still vacancies and defects remaining and trapped inside the oxide structures that form at lower temperatures or even at ambient conditions, leading to relatively inferior mechanical properties of the TiN materials.^41–44^

#### 3.2.2 Compositional Studies Using XPS

XPS Survey scan spectra were recorded over a binding energy (BE) range from −5 to 700 eV with an energy step width of Δ*E* = 0.5 eV. The survey of Ar^+^ ion-sputter-cleaned samples of both TiNO nanowires and thin films showed core-level lines originating from Ti (Ti 2s, Ti 2p, Ti 3s, Ti 3p), N 1s, O 1s, and C 1s. The presence of O 1s peaks at 530.6–531.4 eV highlighted the presence of oxynitride and oxide species (TiNO/TiO_2_) in the samples. High-resolution spectra of Ti 2p, O 1s, and N 1s core-level XPS peaks were recorded. The fitting procedure enabled evaluation of the signals by determining peak positions, heights, and full width at half-maximum; the values and their species assignments are listed in Table S1. To represent the entire Ti 2p (450–470 eV) spectrum, ten different peaks have been fitted using constraints that are based on the physical principles of these peaks. Fitting parameters for Ti 2p spectra were developed using data from the NIST XPS database^45^ and selected literature refs 45–48 . Adventitious carbon, C 1s set at 284.8 eV, was used as an internal standard for binding energy calibration, and in this case, the quoted binding energies are allowed to vary by ± 0.1–0.2 eV following the uncertainty with this method.^48^ A detailed representation of the different contributing species is shown in Figure 3a–c. Three peaks are identified, each spin–orbit split into doublets with j values of 1/2 and 3/2. The peak positions at 455.1, 456.5, and 458.79 eV, identified as (TiN 2p_3/2_, TiNO 2p_3/2_, and TiO_2_ 2p_3/2_ respectively, matched well with the literature.^24,31,47–51^ The gap between the 2p-doublet peaks (2p_3/2_ and 2p_1/2_) of TiN, TiNO, and TiO_2_ remained nearly constant with an average of 5.9, 5.8, and 5.7 eV for TiNO thin film and nanowire. The peak marks the signature of nitride species at 455.1 eV,^50^ while the peak at 456.5 eV indicates the presence of intermediate species of Ti bonded concomitantly to N and O. The formation of oxynitride takes place due to the presence of residual oxygen in the chamber or due to oxygen impurity as in the source nitrogen, which is used during TiN/TiNO nanowire and thin film formation. In our earlier studies, we reported that both TiNO and TiN have a rock-salt crystal structure.^52^ However, the two compounds differ in their lattice constant. The 458.79 peak in Figure 3a has been attributed to oxidized species of Ti–O.^45^ The 397.85 eV peak in the N 1s core-level spectra (Figure 3b) reflects the presence of N–Ti species, whereas the peak at 396.83 eV confirms the presence of the above-mentioned Ti–N–O species. As noted in Figure 3b, the presence of adsorbed (atomic) N–O has also been detected at ∼398.9 eV. The peaks in the O 1s spectra (Figure 3C) at 530.43, 531.2, and 532.75 eV have been assigned to the oxide bond (Ti–O), the oxynitride bond (Ti–N–O), and the chemisorbed N–O bond at 532.75 eV, respectively. In addition to the three main peaks associated with the presence of Ti–N, Ti–O, and Ti–N–O, we have also detected the presence of two plasmonic peaks (Figure 3a), one at 457.9 e V for TiN and the other at 459.55 e V for TiNO, both of which arise due to the spin–orbitals coupling in the two compounds.^24,52^

**Figure 3:**
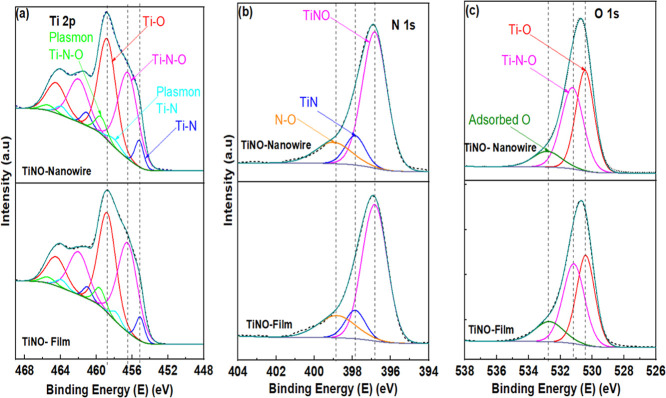
XPS spectra of (a) Ti 2p, (b) N 1s, and (c) O 1s for TiNO nanowires (top panel) and TiNO film (bottom panel). These spectra were recorded after 30 s of Ar + ion-beam etching.

The XPS analysis indicates that the TiNO nanowire and thin film consist of a heterogeneous mixture of TiN, TiNO, and TiO2 phases, coexisting as TiN_*x*_O_*y*_. The surface atomic and phase compositions of the samples, as determined by quantitative XPS, are presented in Table 1. A partial oxidation of TiN to TiNO and TiO_2_ is evident from the areas of the respective XPS peaks. The area value is the widely accepted quantitative measure of phase, as it is directly proportional to the number of atoms of that specific element in a given chemical state (e.g., Ti^3+^, Ti^4+^, N^3–^, O^2–^, etc.) within the sampled volume of the material.^53^ By fitting multiple peaks (components) within a larger spectrum envelope (e.g., the Ti 2p, O 1s, N 1s region), the relative proportions of different chemical states can be determined. As shown in Table 1, the TiNO and TiO2 are present in higher amounts as compared to the TiN phase, confirming the oxidation of TiN to a composite consisting of TiN, TiNO, and TiO_2_ phases.

**Table 1:** Atomic Composition of TiNO Nanowire and TiNO Film from XPS Characterization after 30s Ar^+^ Ion Beam Etching

	titanium 2p (at. %)	nitrogen 1s (at. %)	oxygen 1s (at. %)	relative phase composition (from fitting)		
sample				TiN	TiNO	TiO_2_
TiNO nanowires	34.13 ± 0.15	8.64 ± 0.12	57.23 ± 1.03	4.6%	46.7%	48.3%
TiNO film	34.55 ± 0.23	8.72 ± 0.14	56.73 ± 0.56	4.9%	46.0%	48%

#### 3.2.3 Phase and Orientational Studies Using High Resolution TEM

The atomic structures of TiNO NWs are further resolved by high-resolution scanning transmission electron microscopy (STEM), as shown in Figures S3 and 4. Growing TiNO NWs on the Si substrate allowed us to use a focused ion beam (FIB) to cut a 20 nm lamella to the cross-section of the TiNO NWs. From low-mag STEM images (Figure S3), the TiNO NWs grew on the Si substrate uniformly with random orientation. For most TiNO NWs, there is a small Au nanoparticle on the tip of the NWs, which matches the growth mechanism of TiNO NWs that we propose. A tilt of the TiNO NWs along {110} facet indicates that the crystal structure along (110) of TiNO NWs is almost identical to that of {110} of TiN. TiN has a cubic structure with *a* = *b* = *c* = 4.24 Å, which means that along its = 4.24 Å and *a*’ = a* $\sqrt{2}$ = 6.00 Å. From the high-mag STEM image (Figure 4a), the distance between two Ti atoms along the *c* axis is 4.18 Å, and that along an axis is 2.96 Å, and the angle is 90°. Combining the STEM results with the XRD data, the crystal structure of TiNO nanowires was confirmed to match the rock-salt-like cubic structure of TiN, with the anion O-substituted by N.

**Figure 4:**
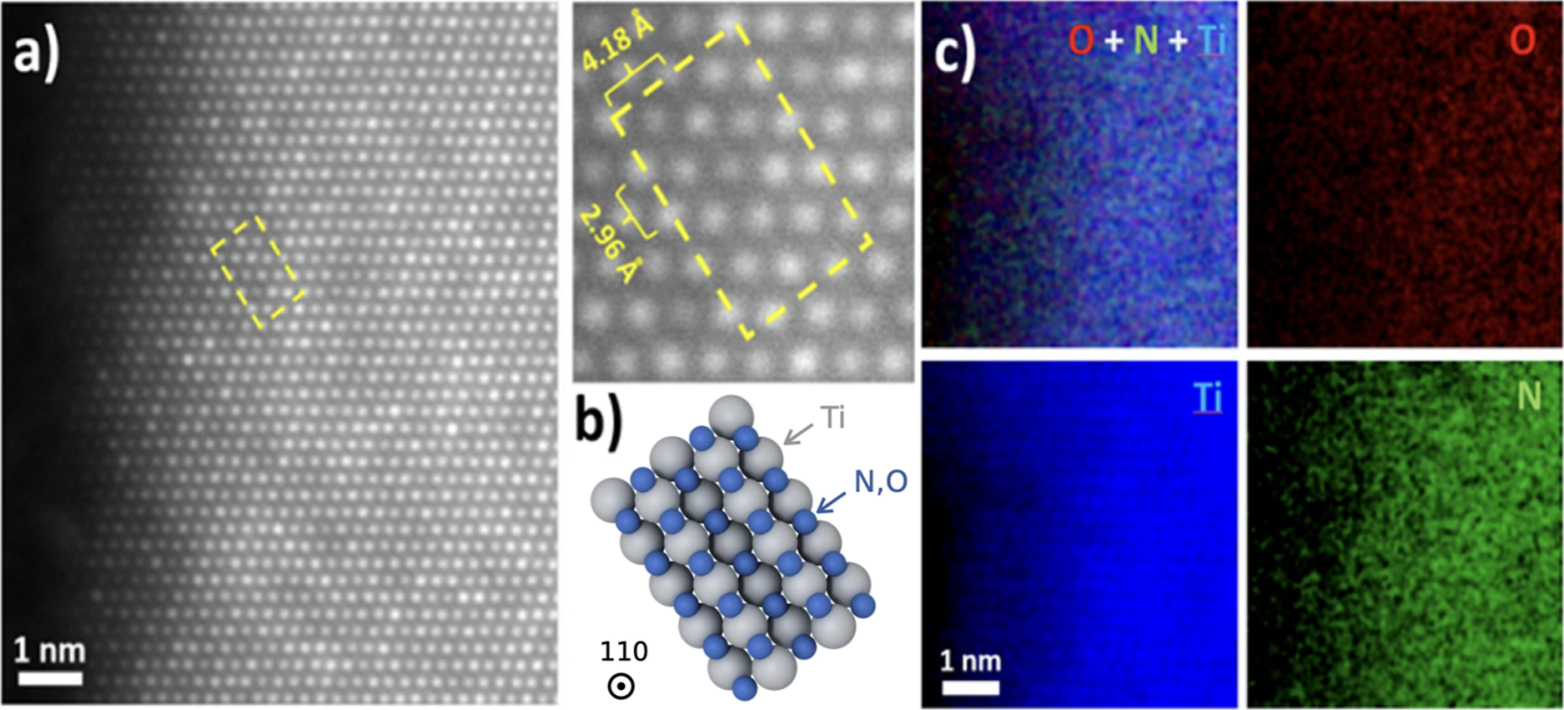
(a) STEM image of the TiNO NW along the direction [110]. (b) Schematic crystal structure of TiNO and (c) EELS spectrum of TiNO NW.

The elemental distribution of TiNO at the atomic scale was analyzed by electron energy loss spectroscopy (EELS), as seen in Figure 4c, and the EELS spectrum of a single TiNO NW, as shown in the annular dark field (ADF) image in Figure S4. We use its Ti L edge, O K edge, and N K edgeto generate the corresponding elemental mapping, respectively. For a single TiNO NW, the Ti, O, and N elements are distributed uniformly in the whole nanowire. More importantly, the TiNO NW does not form an oxide-shell-like structure typical of regular metal nitrides. This phenomenon is further confirmed by a small-field-of-view EELS map at the edge of the TiNO nanowire. Figure S4 clearly shows that Ti has an atomic-resolution EELS map, with O and N uniformly distributed from the core to the shell. The uniform distribution of Ti, N, and O detected through EELS implies a homogeneous oxynitride phase within the entire nanowire volume, rather than a core–shell structure. This homogeneity might promote consistent charge storage kinetics across the entire electrode material.

#### 3.2.4 First-Principles Surface Phase Diagram

To examine the impact of growth conditions on the composition and preferred crystalline orientations of TiNO NWs, we performed density-functional theory (DFT) calculations of surface energies as a function of the titanium, nitrogen, and oxygen chemical potentials. Total energies were calculated using the open-source Quantum-ESPRESSO software, which implements the plane-wave pseudopotential method.^54–56^ The generalized-gradient approximation with the Perdew–Burke–Ernzerhof (PBE) parametrization was used for describing electron–electron interactions, along with pseudopotentials from the SSSP Efficiency library (version 1.3.0) for electron–ion interactions.^57,58^ Kinetic energy and charge density cutoffs of 60 and 600 Ry were selected, respectively. The Brillouin-zone sampling for the electronic ground-state calculations used a shifted 2 × 2 × 1 Monkhorst–Pack *k*-point mesh.^59^ Starting from the Ti_4_N_2_O_2_ rock-salt conventional cell, with N and O arranged in a checkerboard pattern, atomic positions and cell parameters were optimized until all forces on atoms were less than 10^–3^ Ry Bohr^–1^. Symmetric slabs with different crystalline orientations, up to a maximum Miller index of two, were then generated using Pymatgen capabilities.^60^

At fixed temperature, pressure, and chemical potentials, the most stable surface is that with the lowest surface energy, where the surface energy of a given slab, γ, is defined as

4 $$\gamma =\frac{1}{2A}\left(E-TS+PV-\sum_{\alpha }\mu_{\alpha }N_{\alpha }\right)$$

In eq 4, *E* is the total energy of the slab containing two symmetric surfaces of area *A*. The contribution of the entropy term, TS, is neglected, and, under deposition conditions, the PV term is negligible.^61,62^ The surface energy is therefore evaluated as

5 $$\gamma =\frac{1}{2A}\left(E-\mu_{Ti}N_{Ti}-\mu_{N}N_{N}-\mu_{O}N_{O}\right)$$

where *N*_*X*_ is the number of atoms of type *X* in the slab. To prevent surface atoms from forming metallic titanium crystallites or gaseous phases, the chemical potentials of titanium, nitrogen, and oxygen must satisfy μ_Ti_ ≤ *g*_*Ti*_^bulk^, $\mu_{N}\leq g_{N_{2}}^{gas}/2$ , and $\mu_{O}\leq g_{O_{2}}^{gas}/2$ . The Gibbs free energy of titanium is evaluated as the total energy per atom in the *P*6/*mmm* hexagonal bulk structure, while nitrogen and oxygen Gibbs free energies are computed using $g_{X_{2}}^{gas}=E_{X_{2}}^{vacuum}-TS_{X_{2}}^{gas}+E_{X_{2},corr}$ , where $E_{X_{2}}^{vacuum}$ is the calculated energy of the *X*_2_ molecule in a vacuum, $S_{X_{2}}^{gas}$ is the entropy of gaseous *X*_2_,^63^ and $E_{X_{2},corr}$ is a corrective term equal to zero for nitrogen and 1.36 eV for oxygen.^64^ Chemical potentials are further constrained by the thermal equilibrium between the slabs and their corresponding bulk phase, expressed as

6 $$\mu_{Ti}+{0.5\mu }_{N}+0.5\mu_{O}=g_{TiN_{0.5}O_{0.5}}^{bulk}$$

In eq 6, the Gibbs free energy $g_{TiN_{0.5}O_{0.5}}^{bulk}$ is the total energy per formula unit of bulk TiN_0.5_O_0.5_. Denoting -Δ*g* the Gibbs formation energy of bulk TiN_0.5_O_0.5_, and Δμ_Ti_ = μ_Ti_-*g*_Ti_^bulk^, Δμ_N_ = μ_N_-*g*_N_^gas^, and Δμ_O_ = μ_O_-*g*_O_^gas^, then the ranges of chemical potential values are given by -Δ*g*≤Δμ_Ti_ ≤ 0; −2Δ*g*≤Δμ_N_ ≤ 0; and −2Δ*g*≤Δμ_N_ ≤ 0. Within these ranges, the lowest calculated surface energies can become negative, indicating that bulk TiN_0.5_O_0.5_ is unstable. In such cases, we examine possible decomposition into bulk TiN or bulk TiO_2_ by checking whether μ_Ti_+μ_N_ > *g*_TiN_^bulk^ or $\mu_{Ti}+2\mu_{O}>g_{TiO_{2}}^{bulk}$ .

The stability regions of the various TiN_0.5_O_0.5_ surface orientations and terminations are shown in Figure 5. At high nitrogen or oxygen chemical potentials, the surface becomes unstable, favoring the formation of TiN or TiO_2_. At intermediate chemical potentials, surfaces with (111), (110), and (100) orientations are stabilized, depending on thermodynamic conditions. These results are consistent with X-ray diffraction patterns reported in the literature, which show stable (111) TiNO surfaces coexisting with bulk TiN and TiO_2_ in titanium oxynitride thin films.^31,50^ They also suggest that the (110) orientation identified in the present study from STEM imaging (see Figure 4) and XRD (see Figure S2) corresponds to an oxygen-terminated surface, consistent with the high oxygen-to-nitrogen ratios measured by surface-sensitive XPS analysis (see Figure 3 and Table 1). XRD results reported in Figure S2 also indicate that the (110) orientation is more prevalent in the nanowire samples than at the surface of the thin-film samples. Since the (110) surface has a low packing fraction compared to the (111) surface, nanowires may exhibit additional charge storage via ion intercalation (see eq 7 and electrochemical results below). A more detailed investigation of the catalytic nanowire growth mechanism and the interfacial energies governing the equilibrium at the triple-phase line would be required to further interpret the nanowire growth mode and the prevalence of the (110) orientation.

**Figure 5:**
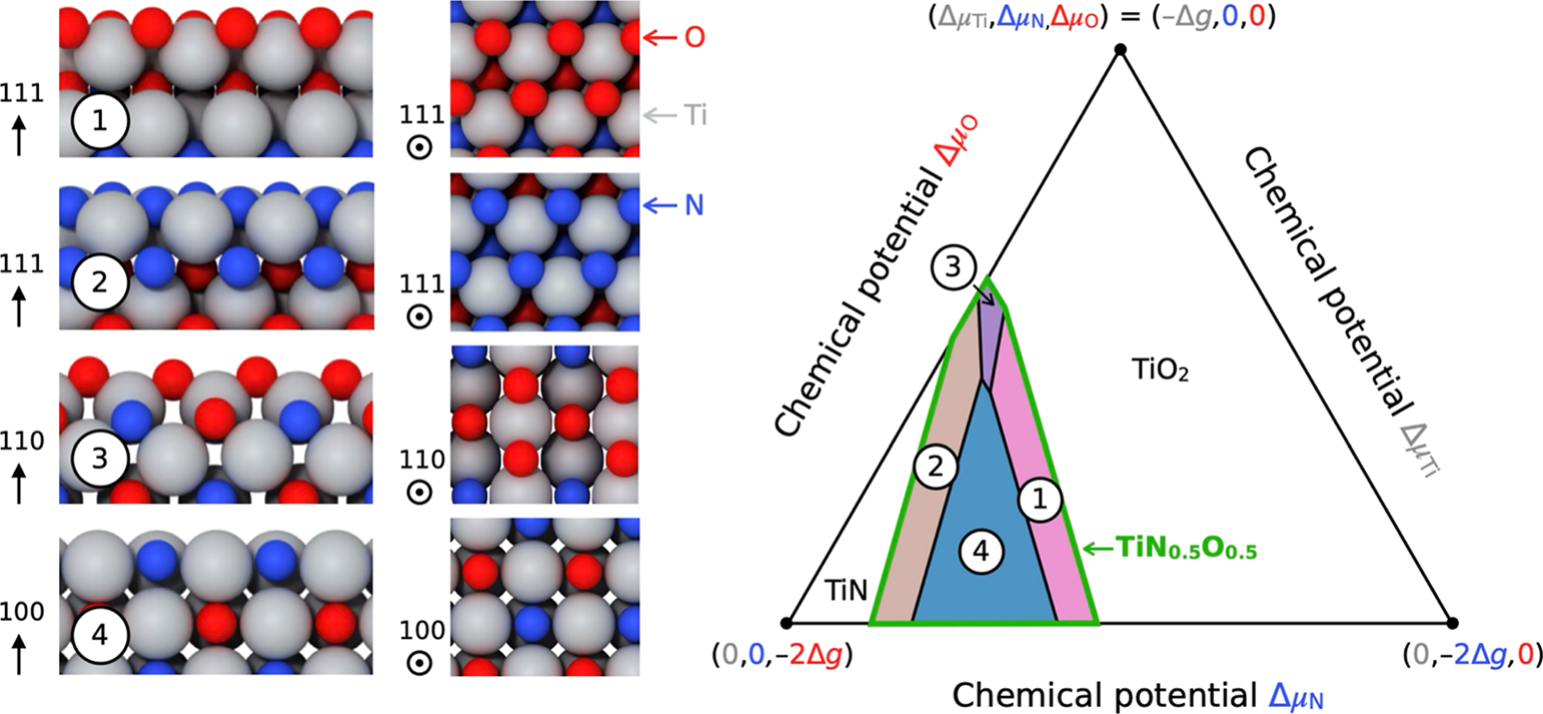
Atomistic models of the (111)-O, (111)-N, (110)-O, and (100) surfaces are shown from side and top views (left panel). Surface phase diagram of TiN_0.5_O_0.5_ as a function of the per-atom chemical potentials of its constituent elements, at room temperature (right panel). The energy -Δ*g* is the Gibbs formation energy of bulk TiN_0.5_O_0.5_, which is calculated to be of −4.24 eV per formula unit. The TiN_0.5_O_0.5_ surface is metastable within the region enclosed by the green line, while it may decompose into the TiN or TiO_2_ phases in the other regions.

### 3.3 Electrochemical Studies for Supercapacitor Applications

#### 3.3.1 Cyclic Voltammetry-Assisted Capacitance Measurements

The cyclic voltammetry (CV) curves of TiNO thin-film and TiNO nanowires grown on silicon (100) substrates are shown in Figure 6a,b, respectively. The test was carried out in 1 M KOH at scan rates from 2 mV/s to 300 mV/s. The TiNO samples exhibit a nonlinear relationship between the current density and scan rate. The nonlinear CV behavior suggests that the TiNO materials system is a pseudocapacitor-type, as opposed to the rectangular-shaped CV curves of the electrical double-layer capacitor materials.^4^ The charge storage mechanism is attributed to the diffusion-controlled redox (Faradaic) reaction between the electrode material surface and electrolyte. The faradaic reaction on the oxidized TiN_*x*_O_*y*_ is expressed as the following reaction eq 7:^65^

7 $${\left({TiN}_{x}\;O_{y}\right)}_{surface}+K^{+}+e^{-}\;\begin{array}{l} Charging\\ ⇌\\ Discharging \end{array}{\left({TiN}_{x}^{-}K^{+}O^{y}\right)}_{surface}$$

**Figure 6:**
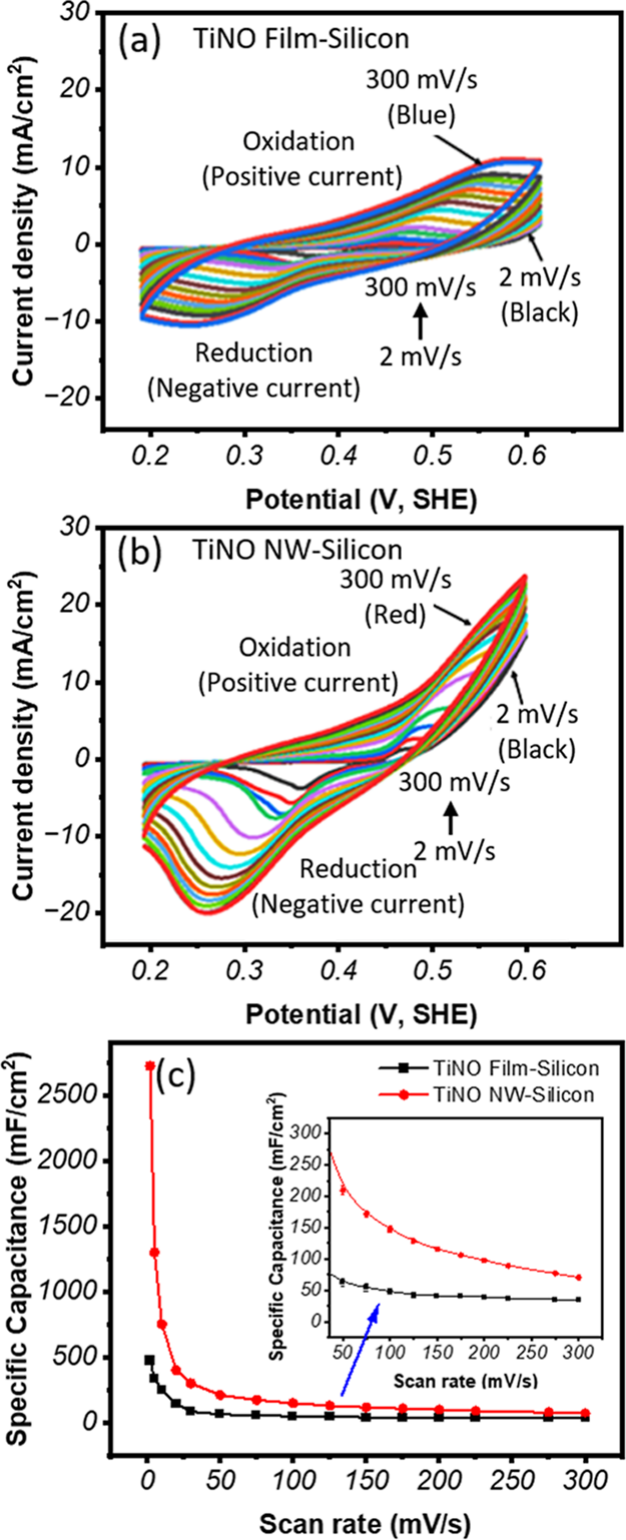
CV curves for (a) TiNO film and (b) TiNO nanowires at the scan rates from 2 mV/s to 300 mV/s, and (c) specific capacitances versus scan rate (mV/s) for TiNO thin-film and nanowire samples; the inset shows a magnified view along *x*- and *y*-axes.

Due to its pseudocapacitive behavior, TiNO electrodes enable reversible surface- or near-surface Faradaic reactions. This feature allows TiNO-based capacitors store more charge. The charge-ion transfer is faster in pseudocapacitors than in materials with the electrical double-layer capacitor behavior, which is based on the physical adsorption/desorption of the electrolyte ions onto the surface of electrode materials.^2,4^ Consequently, TiNO-nanowire materials are expected to surpass the capacity limitations of electrical double-layer capacitors and the mass transfer limitations of batteries,^2,4^ as confirmed by the outstanding capacitive results presented. The areal specific capacitances for the TiNO thin films and TiNO nanowires, obtained using the areas (∫*IdV*) under the CV curves (Figure 6a,b) and eq 8,^65^ are plotted in Figure 6c as a function of applied potential scan rate. This figure shows that the areal specific capacitance of the TiNO nanowire samples is higher than that of the TiNO thin film samples.

8 $$C=\frac{\int IdV}{\Delta V^{*}scanrate}$$

where *I* is the current density (mA/cm^2^), V is the potential (V), Δ*V* is the potential window (V), and scan rate (mV/s), respectively. At a scan rate of 2 mV/s, TiNO nanowires sample on silicon substrate can achieve an aerial specific capacitance value of 2725 mF/cm^2^, which is one of the highest values reported among the recent top-tier nanoscale electrode materials (e.g., vanadium nitride nanosheets/carbon nanotube fibers (564 mF/cm^2^),^66^ W_2_N (550 mF/cm^2^),^67^ N_3_N (319.5 mF/cm^2^),^68^ and ALD-TiN/carbon nanotube (81 mF/cm^2^)^69^). Furthermore, its capacitance is higher than that of the top-record titanium oxynitride hollow nanotubes (1.5 μm length, 120 nm diameter) with the areal capacitance value of 2500 mF/cm^2^ at 2 mV/s under the 1 M KOH electrolyte.^38^ It has also been reported that TiN nanosheet arrays grown on Ti foil have demonstrated specific capacitance on the order of ∼81.6 F/g, with ∼75% retention after 4000 cycles.^70^ Porous TiN paper electrodes have also been found to exhibit extremely high electronic conductivity (∼3.7 × 10^4^ S/m), enabling ultrafast charging in a 1.5 V window and negligible capacitance decay over 200,000 cycles.^71^ There are also reports that surface-engineered TiO_2_ nanorod arrays show a specific capacitance of ∼57.6 mF/cm^2^ in 2 M KOH, with ∼83% rate capability retention at 200 mV/s and ∼91% retention over 10,000 cycles.^72^

It can be seen that the areal-specific capacitance of the TiNO nanowires is improved by the ∼6-fold increase with respect to the TiNO thin film (400 mF/cm^2^) at 2 mV/s. Even at a high scan rate of 300 mV/s, the TiNO nanowires (66 mF/cm^2^) exhibit ∼93% improvements relative to that of the TiNO film (34.18 mF/cm^2^). The improvement in the capacitive properties of the TiNO nanowire samples is attributed to more favorable charge transfer and ion diffusion, owing to the high effective surface area and density of our TiNO nanowire architecture compared with the TiNO thin film. As nanostructuring increases the specific surface area, the interfacial contact area between the electrode and the electrolyte also increases, leading to higher capacitance and substantially shorter diffusion distances for ions from the electrolyte to the redox sites in energy storage of TiNO nanowires.

The coexistence of TiN, TiNO, and TiO_2_ phases in a composite material enhances charge storage via synergistic effects.^73,74^ High-conductivity metallic components, such as the TiN phase, allow electron flow within the material and to the current collector with low resistance. The metallic character improves supercapacitors’ high-rate capabilities, conductivity, and power density. Semiconducting titanium dioxide is crucial for charge storage due to pseudocapacitive mechanisms that include ion intercalation and deintercalation, such as K^+^, within its crystal structure or surface defects. This process provides high energy density but is often diffusion-controlled and slower than surface capacitance. TiNO and its suboxides (TiO, Ti_2_O_3_) may transition between the highly conductive TiN and the ion-storing TiO_2_. The change in chemical states (Ti^4+^, Ti^3+^, Ti^2+^) creates a heterojunction with favorable band alignment, maximizing charge carrier transmission and minimizing charge recombination. Composite structures take advantage of each component. The TiN provides the conductive backbone, the TiO_2_ offers significant capacity through pseudocapacitation/intercalation, and the TiNO interfaces enable smooth charge transfer between the phases. This creates a dual-storage mode system that combines the high-power density of conductive material with the high energy density of intercalation material, improving electrochemical performance compared to any single phase alone. Nanowires further boost charge storage due to a higher surface area. At this point, it is worth mentioning here that Au nanodots located on top of each TiNO nanowire may promote localized charge transfer and affect the electrochemical properties of the TiNO nanowires in a very confined region, as the size of the Au nanodots (∼10 nm) is much smaller than the length of the TiNO nanowire (420 nm). However, presently, we are not able to assess/estimate quantitatively the contribution of Au nanodots to the electrochemical properties of the TiNO nanowire samples.

Larger values of anodic current densities around −0.27 V and higher capacitance values can be understood using a schematic diagram shown in Figure S1. As shown in Schematic (Figure S1), the pseudocapacitance in our TiNO thin film and nanostructure is attributed mainly due to Faradaic redox reactions with a small contribution from the non-Faradaic region. The non-Faradaic contribution is stronger in the TiNO thin film samples as reflected from the near rectangular shape of the CV curves. As shown in this schematic, the TiNO thin-film sample has a smaller surface area than the TiNO nanowire sample for surface redox reactions. The schematic also shows that charge transfer in the redox reaction is more efficient along the nanowire’s diameter due to the nanowire’s nearly one-10th dimension. The diameter of the nanowire is ∼20 nm, while the thickness of the TiNO films is ∼70 nm. Thus, the larger cumulative surface area provided by the high density of TiNO nanowire samples, along with more efficient charge transfer within individual nanowires, results in higher anodic current densities and specific capacitance values for the TiNO nanowire samples.

A linear dependence of the peak current density on the square root of the scan rate (ν^0.5^) or directly on the scan rate (ν^1^) has been used to ascertain if the charge transfer mechanism is diffusion-controlled or adsorption-controlled. As shown in Figure S5, the current density fits better with the square root of the scan rate than with the linear scan rate. This is evident from the value of the coefficient of determination (*R* ^2^), which quantifies how well the regression model explains the variability of the experimental data. A higher *R* ^2^ value indicates a better fit between the model and the observed results. In our case, the *R* ^2^ value for the current density-square-root scan rate plot is 99%, significantly higher than the 93% obtained for the linear scan rate plot, confirming that a diffusion-controlled mechanism more strongly governs the electrochemical process. It is also evident from Figure S5 that each data set can be divided into two regions, each having different values of b. The validity of the square root fitting extends to a larger range than the linear fit of the scan rate. Based on this analysis, we infer that the charge-transfer mechanism is predominantly diffusion-controlled.

#### 3.3.2 Galvanostatic Charge and Discharge Measurement

The electrochemical studies involving the decay of capacitor potentials were conducted at different current densities as a function of time to understand the discharging behavior of TiNO thin film and nanowire, and the result is shown in Figure 7a,b. From the discharge curve, the areal-specific capacitance values are determined by the equation^38,65^

9 $$C=\frac{I^{*}\Delta t}{\Delta V^{*}S}$$

where *I* is the discharge current (*A*), Δ*t* is the discharge time (s), Δ*V* is the potential window (V), and *S* is the surface area of the working electrode (cm^2^), respectively. It is evident from Figure 7c that the TiNO nanowire sample (areal capacitance of 143 mF/cm^2^ at 0.5 mA/cm^2^) has a significantly improved charge storage (∼3.4-fold) as compared to that of TiNO thin film (36 mF/cm^2^) at the same current density. These capacitance results from discharge measurements are also greater than those reported in the literature for many materials (e.g., Nb_4_N_5_ nanobelt powders (37.4 mF/cm^2^ at a current density of 0.2 mA/cm^2^),^75^ hydrogenated TiO_2_ nanotube arrays (4.64 mF/cm^2^ at the current density of 0.1 mA/cm^2^)^76^ and graphene-carbon nanotube electrode (32.6 mF/cm^2^ at the current density of 0.1 mA/cm^2^).^77^ The capacitance of our samples is also expressed as the mass-specific capacitance (F/g) that removes the ambiguity about using the actual nanowire surface area or the substrate area over which the nanowire samples are grown. Using the reported^78^ TiNO density of ∼5.4 g/cm^3^ and estimated total mass of ∼8.6 μg for TiNO nanowires and ∼8.3 μg for TiNO films, the specific capacitances of the TiNO nanowire and thin film samples have been found to be in the range of 3340 F/g to 1479 F/g and 871 F/g to ∼473 F/g, respectively, with the variation of the current densities from 0.5 mA/cm^2^ to 5 mA/cm^2^. These calculations are based on the number of the nanowires ∼20 wires per 200 nm × 200 nm area as shown in Figure 7d, and their dimension of ∼420 nm in length, ∼21.4 nm in diameter. The thin film samples were ∼70 nm thick. Since the TiNO nanowires are grown on rigid substrates, the conventional Brunauer–Emmett–Teller (BET) analysis is impractical and nonrepresentative of the accessible surface area during electrochemical operation. The surface area measurements of the TiNO nanowire sample obtained using FE-SEM and ImagePro are considered sufficiently accurate for the present study. We have also tested the TiNO thin films in a two-electrode supercapacitor device. In this device, a symmetric supercapacitor device was fabricated using two TiNO thin-film working electrodes. The two electrodes were separated by a chromatographic paper soaked in 0.1 M KOH for 30 min. The surface area for both positive and negative electrodes was 1 cm × 1 cm. The device structure and results obtained are shown in Figure S8. The supercapacitance (∼84 mF/cm^2^ at 1.0 mA/cm^2^) and discharge time (84 s) of this sample are comparable to those measured in a three-electrode electrochemical cell, validating the device potential of the TiNO materials system.

**Figure 7:**
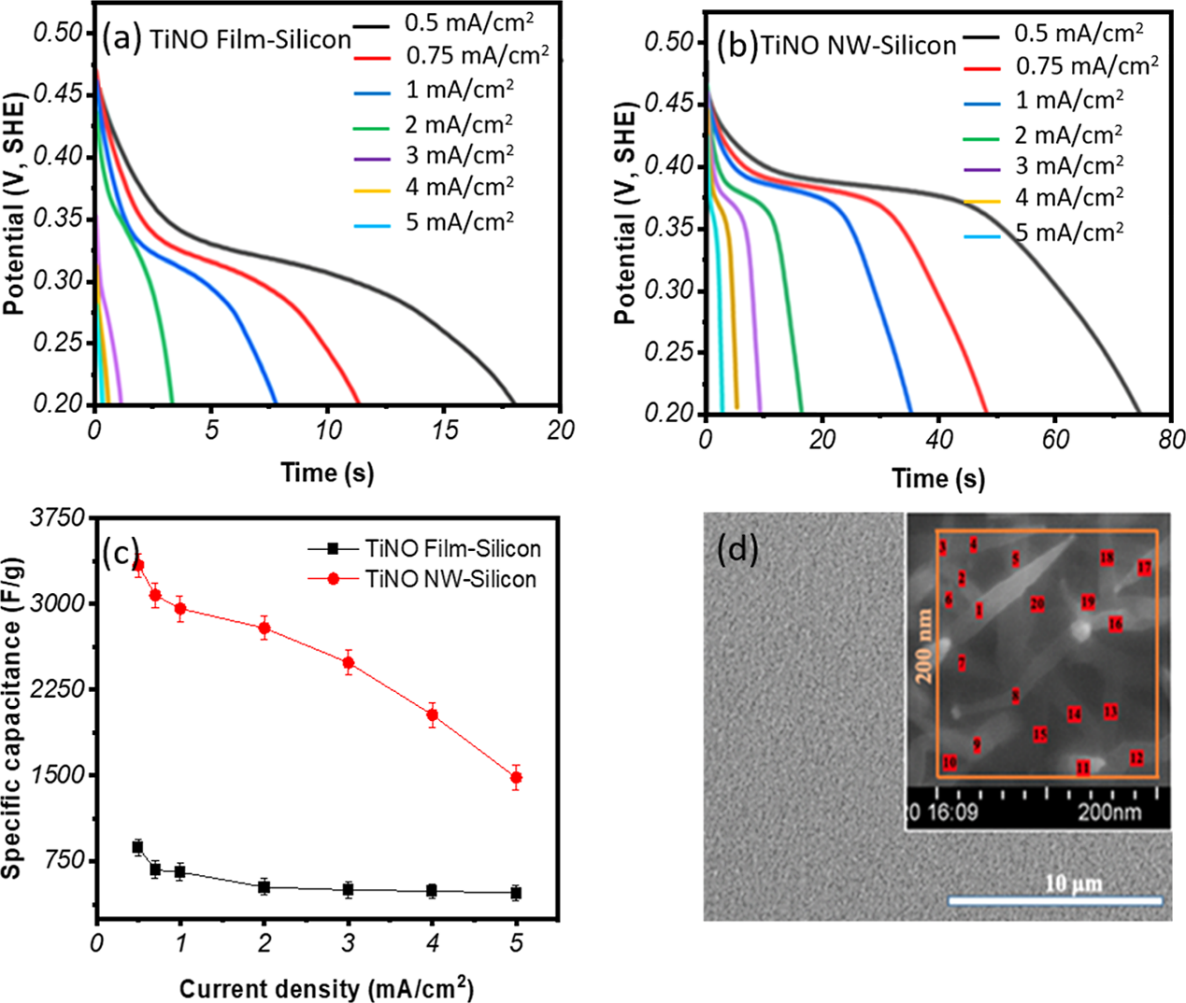
Discharge curves of (a) TiNO film and (b) TiNO nanowire samples recorded at different current densities, (c) specific capacitance versus current density for TiNO thin-film and TiNO nanowire samples, and (d) FE-SEM images displaying a high-density number of the TiNO nanowires at two magnifications.

At the outset, the TiNO thin-film electrodes seem to have better rate-performance, as indicated by a slower decrease in specific capacitance with increasing sweep rate of the applied potential (Figure 6c) and current density (Figure 7c). However, a plot of normalized capacitance as a function of sweep rate (Figure S7a) shows that both the TiNO nanowire and the thin-film samples have similar rate performance. The same inference is obtained when a log–log plot of specific capacitance versus scan rate (Figure S7b) and a log–log plot of specific capacitance versus current density (Figure S7c) are created. The slopes of these plots, which are taken as the rate constants of charge transfer reactions, for the nanowire and thin film samples, are similar. However, a marginal difference in rate performance between TiNO thin-film and TiNO nanowire samples may be rooted in the nature of the CV curves for the two sets of samples. As noted in Figure 6a,b, the pseudocapacitance behavior (nonrectangular shape of CV curves) in the TiNO nanowire samples is more protuberant than for the TiNO thin film samples (near-rectangular shape of the CV curves that is typically observed in EDLCs). As EDLCs are characterized by a physical ion adsorption/desorption process and pseudocapacitors by a surface redox reaction, the effect of an increase in the potential sweep rate and current density will be milder on the ion adsorption/desorption process than on the kinetics of the surface redox reactions.^79–81^

#### 3.3.3 Electrochemical Impedance Spectroscopy Studies, Stability, and Ragone Plot

A comparison of the Bode plots, obtained using EIS, for the TiNO nanowire and TiNO thin film samples is illustrated in Figure 8a. The time constant is an important aspect of the supercapacitor materials to interpret the minimum time required to charge and discharge

10 f=1/2πτ

**Figure 8:**
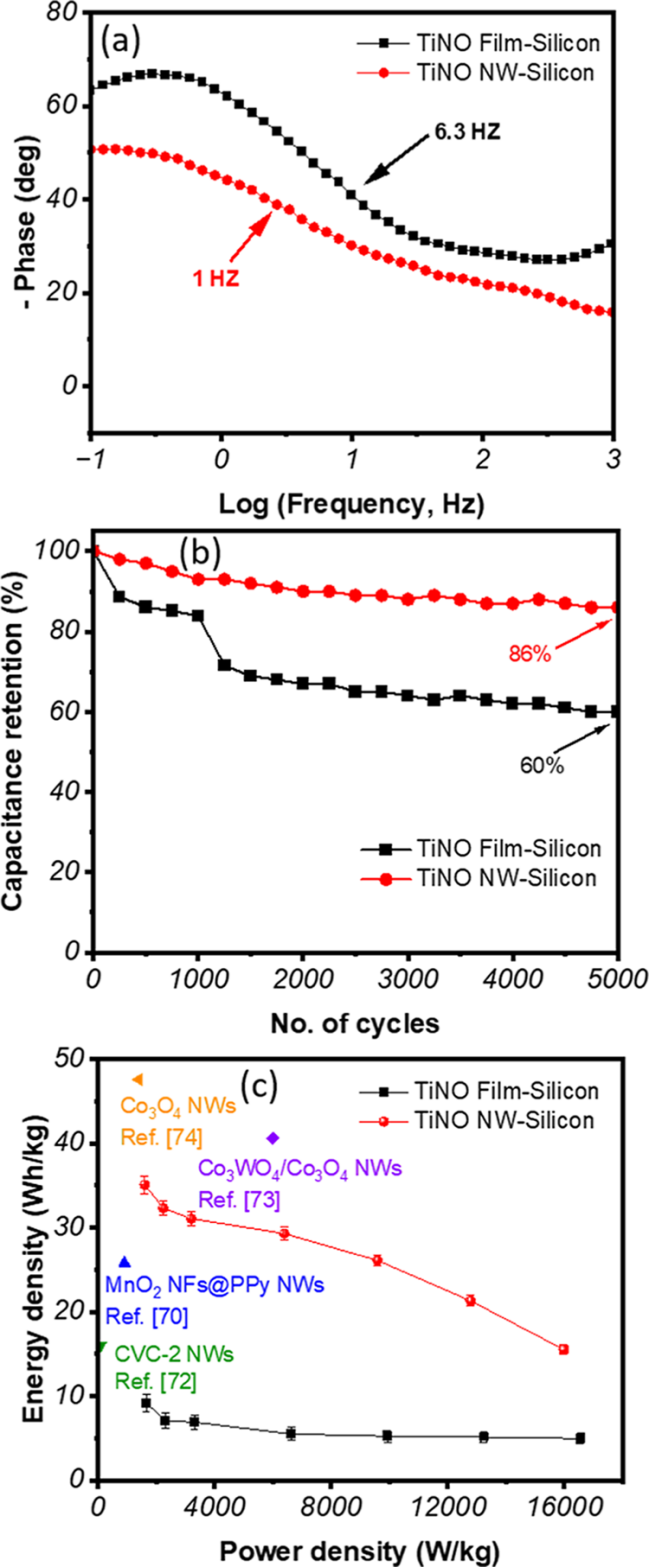
(a) Phase angle versus frequency curves using Bode plots for TiNO thin-film and TiNO nanowire samples, (b) stability cycling performance of the TiNO thin-film and TiNO nanowire samples, and (c) energy density versus power density Ragone plots for the TiNO thin-film and TiNO nanowire samples.

where *f* is a frequency (Hz) at −45° from the Bode plot, and τ is a time constant (*s*). The time-constants of these two samples are determined using the characteristic frequency (*f*) at −45° from Bode plots, which indicate the resistive impedance equal to the capacitive impedance, by substituting the frequency value into eq 10.^4,65,82,83^ From the Bode plots Figure 8a, the characteristic frequencies (*f*) at −45° of the samples are found to be 1 Hz for the TiNO nanowires and 6.3 Hz for the TiNO film sample. The time constant (τ) values are calculated to be ∼0.16 s and ∼0.025 s for the TiNO nanowires and TiNO thin film, respectively. These minimum times required for the charge/discharge of both TiNO samples exhibit a relatively faster time than the normal range of the time constant (1 to 10 s) reported in supercapacitors.^4^ The cyclic stability performance of the TiNO nanowires and thin film samples was performed for 5000 cycles using a fixed current density of 5 mA/cm^2^. It is important to note that pseudocapacitive materials often show significantly greater capacitance values than those for the electrical double-layer capacitor (EDLCs)-type materials. At the same time, pseudocapacitive materials are also marked by their poor cycling performance. For example, Nb_4_N_5_ loses 20% capacitance after 1000 cycles,^75^ and titanium nitride (TiN) (core)/Ni­(OH)_2_ (shell) nanowire arrays lose 75% capacitance after 150 cycles^84^). As seen in Figure 8b, the capacitance retention of the TiNO nanowire sample illustrates promising results, with 86% retention after 5000 cycles, meanwhile, the capacitance retention of the TiNO thin film shows 60% after 5000 cycles. This stability enhancement of our TiNO nanowires sample may come from the additional protective layer of the stable phase rutile TiO_2_, which was typically formed at a high temperature above 700 °C, improving the chemical resistance and, as a result, the stability of the prepared materials can last longer. Furthermore, the areal energy density (E, Wh/cm^2^) and power density (P, W/cm^2^) of the TiNO nanomaterials can be determined by the following eqs 11 and 12.^65,85,86^

11 E=12(C)(ΔV23600)

12 $$\text{and}\;P=\frac{E}{\Delta t}\times 3600$$

where *E* is the areal energy density (Wh/cm^2^), *C* is a real specific capacitance (F/cm^2^), and Δ*V* is the potential window (*V*), Δ*t* is the discharging time (*s*), respectively. As illustrated in the Ragone plot (Figure 8c), the TiNO nanowire sample exhibits an excellent energy density of 35 Wh/kg at a power density of 1598 W/kg. This performance is comparable to the reported values for porous polypyrrole nanowires/manganese oxide nanoflakes (MnO_2_ NFs@PPy NWs), which deliver 25.8 Wh·kg^–1^ at 901.7 W/kg,^85^ and is significantly higher than that of carbon nanotubes and vanadium pentoxide (V_2_O_5_) nanowires, reported at 16 Wh/kg and 75 W/kg, respectively.^87^ Furthermore, cobalt tungsten oxide and cobalt oxide nanowires have shown energy and power densities of 57.8 Wh/kg and 6000 W/kg, respectively,^88^ while cobalt oxide nanowires alone exhibit 47.6 Wh/kg at 1392 W/kg.^89^ These comparisons underscore the competitive performance of the TiNO nanowire electrode. Meanwhile, the TiNO thin film shows an energy density of 9 Wh/kg at a power density of 1598 W/kg. The improvement in energy densities of the TiNO nanowires is based on the enhancement of their higher effective surface area for the charge transfer and ion diffusion at the electrode–electrolyte interface than that of the TiNO thin film. These electrochemical studies have shown that the binder-free titanium oxynitride nanowires prepared by PLD can be a potential candidate as the future electrode material for electrochemical energy storage in supercapacitor applications.

## 4 Conclusions

We have demonstrated that the high capacitive performance TiNO nanowires can successfully be grown on silicon substrates by a pulsed laser deposition method without employing a binder in the materials. Although the Au catalyst is essential for nanowire growth, its direct quantitative contribution to the electrochemical capacitance has not been isolated in this study and is anticipated to be negligible in light of its small surface area in comparison to the large surface area of the TiNO nanowires. With the high effective surface area of the one-dimensional structure for enhancing ion transport at the electrode–electrolyte interface, the TiNO nanowires show superior electrochemical performance compared to the two-dimensional TiNO thin film. TiNO nanowires can achieve remarkable specific capacitances ranging from 2725 mF/cm^2^ to 66 mF/cm^2^ under scan rates from 2 mV/s to 300 mV/s, respectively. The TiNO nanowires also exhibit good capacitance retention and fast charge–discharge capability. These findings could open new opportunities for TiNO-based nanomaterials in future high-performance supercapacitors and other energy storage applications.

## Supplementary Material



## Notes

Figure S1. Schematic representation of ion adsorption behaviors on (a) TiNO thin-film and (b) TiNO nanowire samples. Figure S2: XRD patterns of TiNO nanowires and TiNO film grown on silicon (100) substrates under the same deposition conditions of 800 °C, 12,000 pulses, and 200 mTorr N_2_; the XRD data also include the XRD patterns of gold (Au) nanodots and gold film as well. Figure S3. (a) Low-magnification STEM image of the TiNO nanowires on the Si substrate cut by FIB. (b,c) High-magnification STEM images of a single TiNO nanowire. Figure S4. Representative FE-SEM image used for calculating the growth yield of TiNO nanowires. Figure S5. EELS spectrum of a single TiNO NW containing an annular dark field (ADF) image and O, N, Ti, C mapping. Figure S6. Fitting of peak current density on the square root of the scan rate (ν^0.5^) or directly on the scan rate (ν^1^) of the TiN nanowire sample. Figure S7. Rate-performance behaviors of the TiNO nanowire and TiNO thin-film samples: (a) normalized specific capacitance vs scan rate. (b) Log–log plot of specific capacitance and scan rate obtained from the cyclovoltammetry study, and (c) log–log plot of specific capacitance and current density (obtained from galvanostatic charge–discharge measurement). Figure S8. (a) Schematic of a two-electrode symmetric TiNO supercapacitor, (b) charge–discharge behaviors, and (c) specific capacitance as a function of current density for a TiNO thin-film sample. Table S1. Spectral fitting parameters for Ti 2p and N 1s: binding energy (eV) and fwhm (eV) obtained after fitting. Table S2: summary of calculated data of electrochemical measurements at 275 mV for TiNO nanowire (mass = 8.6 μg, area = 0.2 cm^2^) and TiNO thin film (mass = 8.3 μg, area = 0.2 cm^2^) samples (PDF)
