# Supplementary material for: Pseudocapacitive Titanium Oxynitride Nanowires for Ultrahigh Capacitance Supercapacitors
Source: ACS Appl Nano Mater. 2026 Jan 26;9(5):2269–83. doi: 10.1021/acsanm.5c04882 (PMC12887935; doi:10.1021/acsanm.5c04882)
Supplement: Supplementary file 1 [file an5c04882_si_001.pdf]

Supporting Information

Pseudocapacitive Titanium Oxynitride Nanowires for Ultra-high Capacitance Supercapacitors

Sheilah Cherono[a], Panupong Jaipan[a], Zixiao Shi[b], Simon Gelin[c], Joan Ejeta[d], Ikenna Chris-Okoro[a], Mengxin Liu[a], Ghanashyam Gyawali[a], Wisdom Akande[a], Jonghyun Choi[e], Swapnil Nalawade[f], Shobha Mantripragada[f], Ram K. Gupta[e], James D. Schall[a], Kristen L. Rhinehardt[d], Ismaila Dabo[c], Shyam Aravamudhan[f], Bishnu P. Bastakoti[g], David A. Muller[h], and Dhananjay Kumar[a]*

[a] Department of Mechanical Engineering, North Carolina Agricultural and Technical State University, Greensboro, NC 27411, USA

[b] Department of Chemistry and Chemical Biology, Cornell University, Ithaca, NY 14850, USA

[c] Department of Materials Science and Engineering, and Wilton E. Scott Institute for Energy Innovation, Carnegie Mellon University, Pittsburgh, Pennsylvania 15213, USA

[d] Department of Computational Science and Engineering, North Carolina A&T State University, Greensboro, NC 27411, USA

[e] Department of Chemistry, Kansas Polymer Research Centre, Pittsburgh State University, Pittsburgh, KS 66762, USA

[f] Department of Nanoengineering, Joint School of Nanoscience and Nanoengineering, Greensboro, NC 27401, USA

[g] Department of Chemistry, North Carolina A&T State University, Greensboro, NC 27411, USA

[h] School of Applied and Engineering Physics, Cornell University, Ithaca, NY 14853, USA

*Corresponding author. E-mail address: dkumar@ncat.edu

Content

1. **Figure S1.** Schematic representation of ion adsorption behaviors on (a) TiNO thin-film and (b) TiNO nanowire samples.
2. **Figure S2:** XRD patterns of TiNO nanowires and TiNO thin film grown on silicon (100) substrates under the same deposition conditions of 800 °C, 12,000 pulses, and 200 mTorr N_2; the XRD data also include the XRD patterns of gold (Au) nanodots and gold film as well.
3. **Figure S3.** (a) Low-magnification STEM image of the TiNO nanowires on the Si substrate cut by FIB. (b-c) high-magnification STEM images of a single TiNO nanowire.
4. **Figure S4.** Representative FE-SEM image used for calculating the growth yield of TiNO nanowires.
5. **Figure S5.** EELS spectrum of a single TiNO nanowire containing an annular dark field (ADF) image and O, N, Ti, C mapping.
6. **Figure S6.** Fitting of peak current density on the square root of the scan rate ($\nu^{0.5}$) or directly on the scan rate (ν^1) of the TiNO nanowire sample.
7. **Figure S7.** Rate-performance behaviors of the TiNO nanowire and TiNO thin-film samples: (a) normalized specific capacitance vs scan rate. (b) log-log plot of specific capacitance and scan rate obtained from the cyclovoltammetry study, and (c) log-log plot of specific capacitance and current density (obtained from galvanostatic charge-discharge measurement).
8. **Figure S8.** (a) Schematic of a two-electrode symmetric TiNO supercapacitor, (b) Charge-Discharge behaviors, and (c) Specific capacitance as a function of current density for a TiNO thin-film sample.

9. **Table S1.** Spectral fitting parameters for Ti 2p and N 1s: binding energy (eV) and FWHM (e V) obtained after fitting.
10. **Table S2:** Summary of calculated data of electrochemical measurements at 275 mV for TiNO nanowire (mass = 8.6 μg, area = 0.2 cm^2) and TiNO thin film (mass = 8.3 μg, area = 0.2 cm^2) samples.

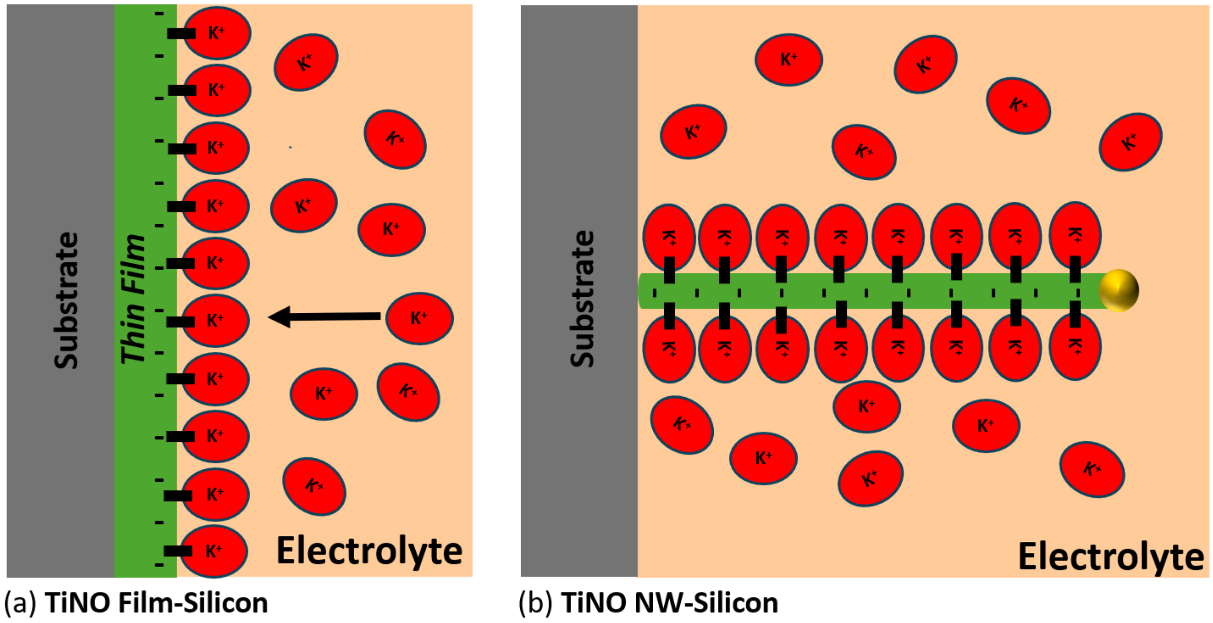

Figure. S1. Schematic representation of ion adsorption behaviors on (a) TiNO thin-film and (b) TiNO nanowire samples.

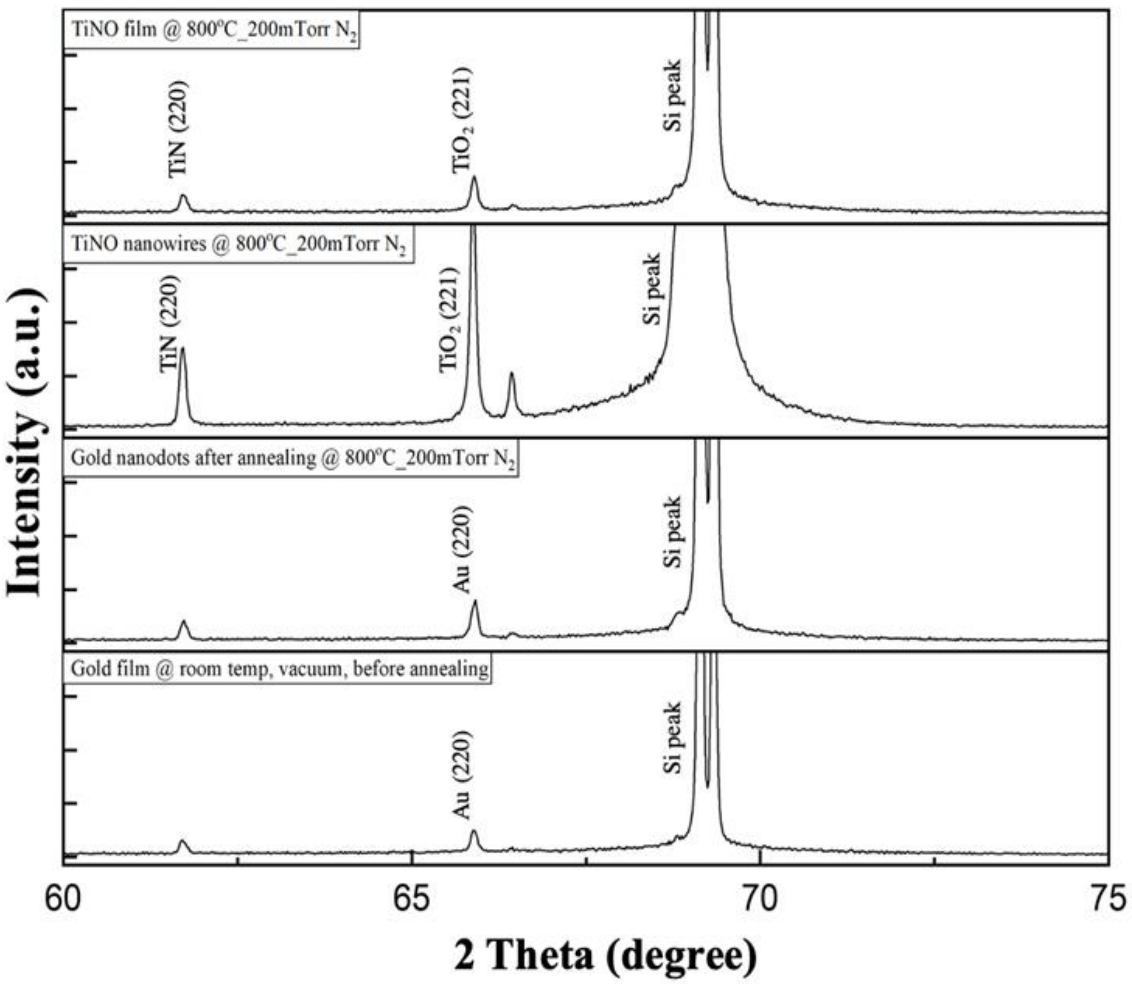

Figure S2. XRD patterns of TiNO nanowires and TiNO film grown on silicon (100) substrates under the same deposition conditions at 800 °C, 12,000 pulses, and 200 mTorr N_2; the XRD data also include the XRD patterns of gold (Au) nanodots and gold film as well.

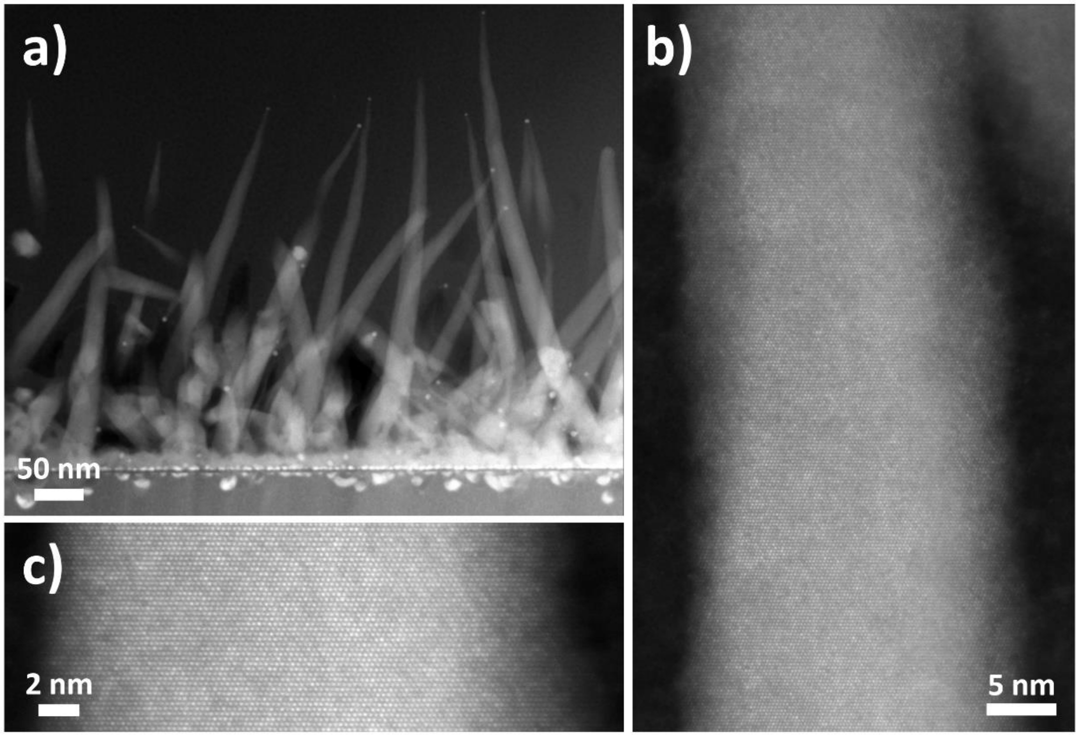

Figure S3. (a) Low-magnification STEM image of the TiNO NW on Si substrate cut by FIB. (b-c) high-magnification STEM images of a single TiNO NW.

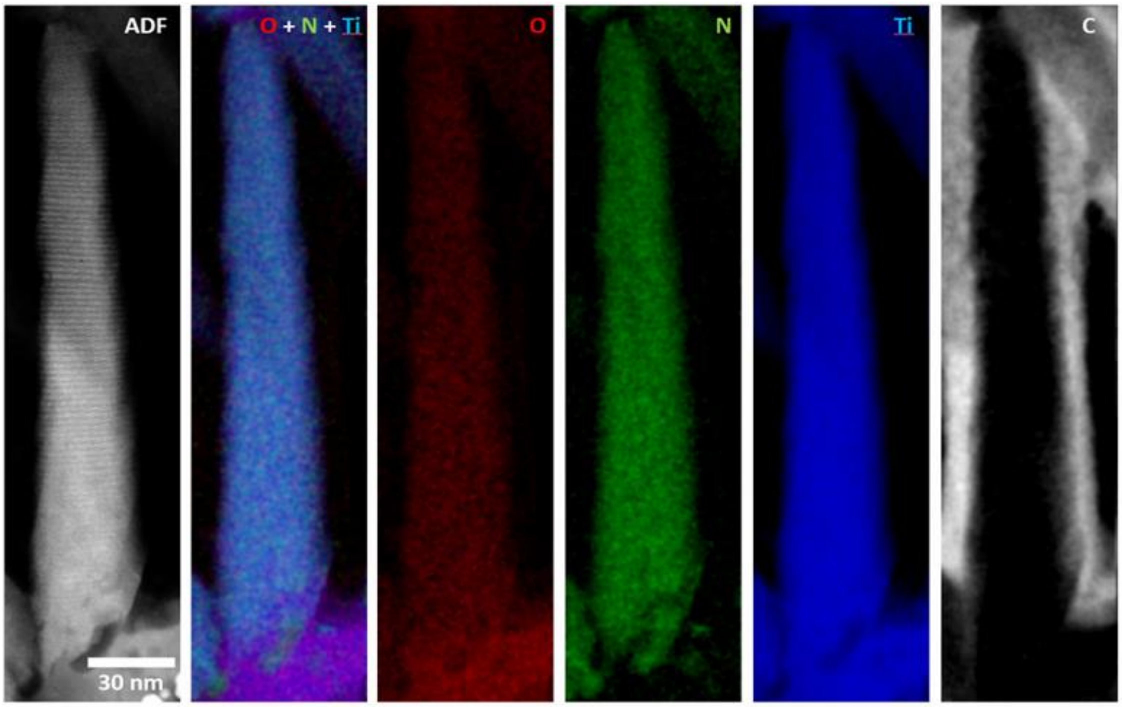

Figure S5. EELS spectrum of a single TiNO NW containing annular dark field (ADF) image and O, N, Ti, C mapping.

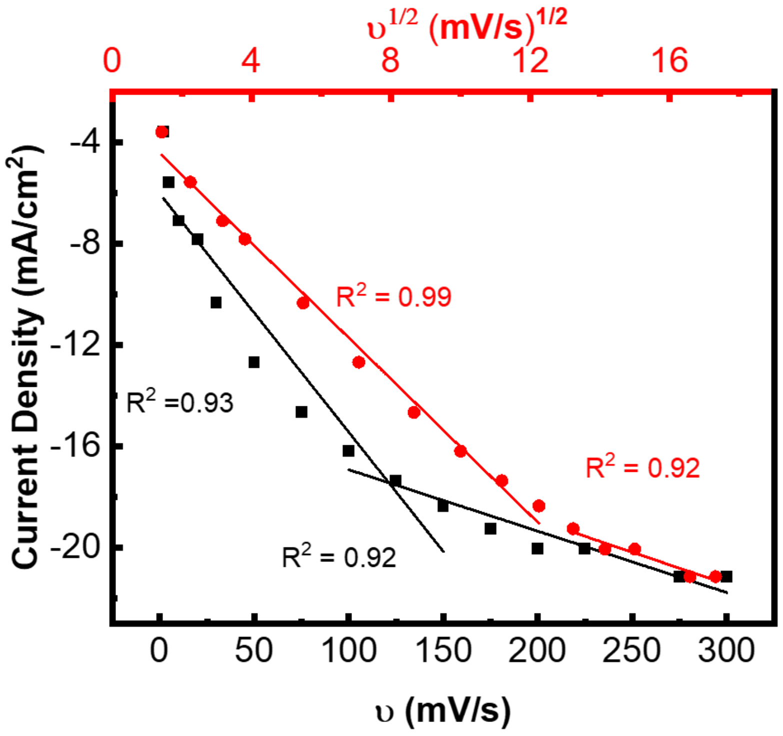

Figure S6. Fitting of peak current density on the square root of the scan rate ($\nu^{0.5}$) or directly on the scan rate (ν^1) of the TiNO nanowire sample.

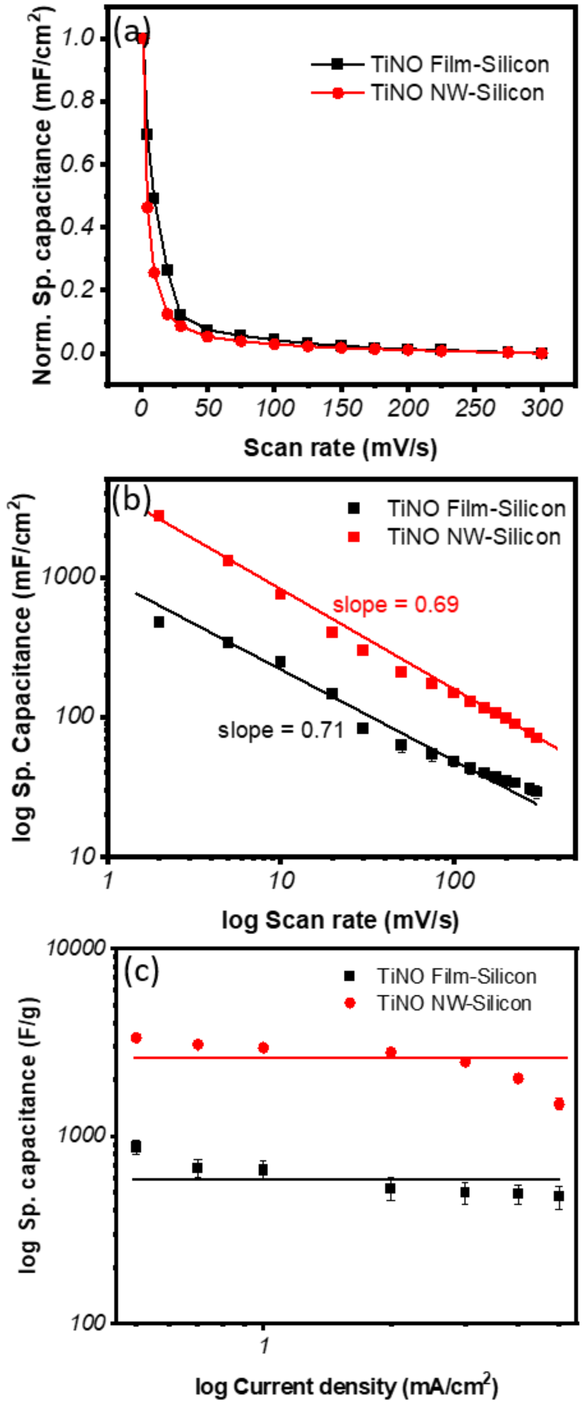

Figure S7. Rate-performance behaviors of the TiNO nanowire and TiNO thin-film samples: (a) normalized specific capacitance vs scan rate. (b) log-log plot of specific capacitance and scan rate obtained from the cyclovoltammetry study, and (c) log-log plot of specific capacitance and current density (obtained from galvanostatic charge-discharge measurement).

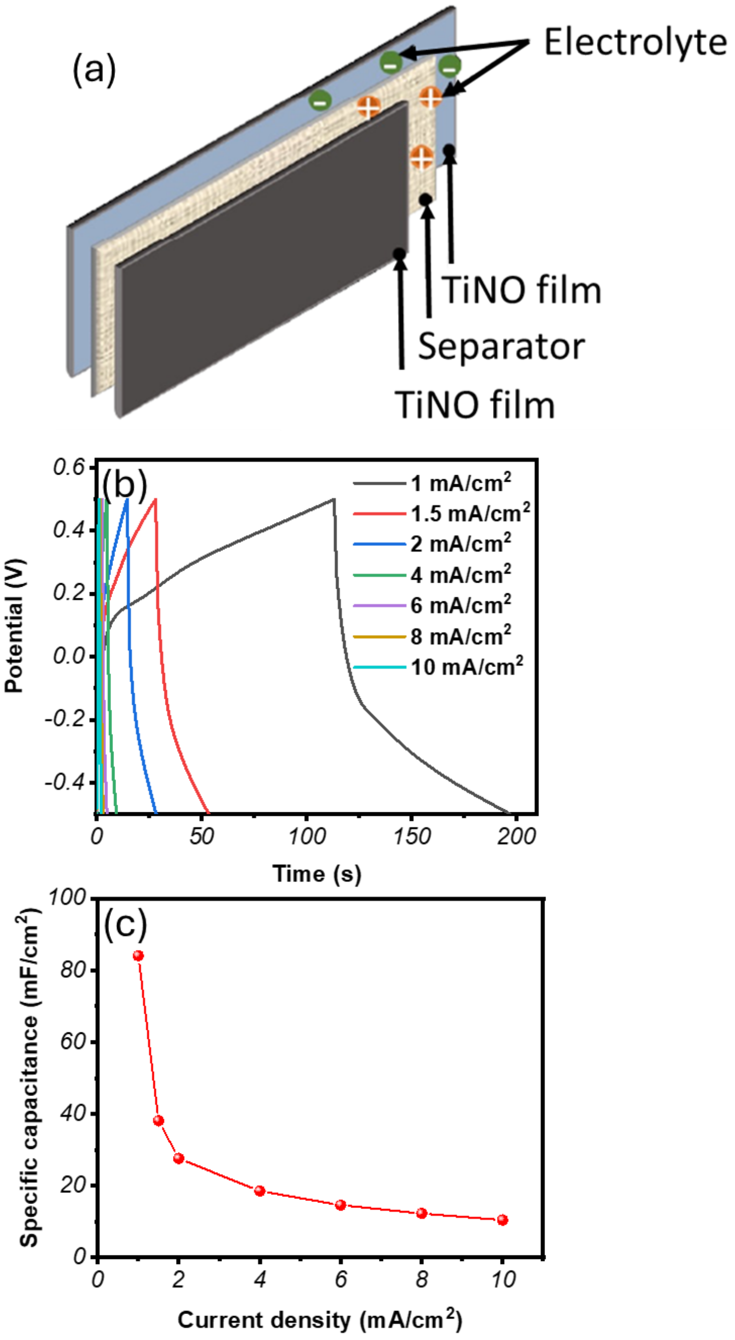

Figure S8. (a) Schematic of a two-electrode symmetric TiNO supercapacitor, (b) Charge-Discharge behavior, and (c) Specific capacitance as a function of current density for a TiNO thin-film sample.

Table S1. Spectral fitting parameters for Ti 2p and N 1s: binding energy (eV) and FWHM (e V) obtained after fitting.

**Assignment**	**Binding Energy (eV)**	**FWHM (eV)**
Ti-N 2p $_{3/2}$	455.1 ±0.1	1.17
Ti-N 2p $_{½}$	461.0 ±0.05	1.17
Ti-N-O 2p $_{3/2}$	456.5 ±0.03	2.7
Ti-N-O 2p $_{1/2}$	462.0 ±0.1	2.7
Ti-O_2 2p $_{3/2}$	458.79 ±0.3	2.38
Ti-O_2 2p $_{½}$	464.49 ±0.2	2.62
Plasmon Ti-N 2p $_{3/2}$	457.9 ±0.1	1.34
Plasmon Ti-N 2p $_{1/2}$	463.9 ±0.05	1.52
Plasmon Ti-N-O 2p $_{3/2}$	459.55 ±0.03	1.54
Plasmon Ti-N-O 2p $_{1/2}$	465.38 ±0.1	1.87
N 1s (N-Ti)	397.85 ±0.1	1.28
N 1s (N-O-Ti)	396.83 ±0.1	1.57
N 1s (N-O)	398.9 ±0.1	2.3
O1s (O-Ti)	530.43 ±0.2	1.27
O1s(O-Ti-N)	531.2 ±0.2	1.7
O1s (N-O)	532.75 ±0.2	2.2

Table S2: Summary of calculated data of electrochemical measurements at 275 mV for TiNO nanowire (mass = 8.6 μg, area = 0.2 cm^2) and TiNO thin film (mass = 8.3 μg, area = 0.2 cm^2) samples.

Current density (mA/cm^2)	Discharge time (s)	Specific capacitance (gravimetric) (F/g)	Specific capacitance (areal) (F/g)	Energy density (Wh/kg)	Power density (W/kg)
TiNO nanowire sample					
0.5	79.0	3340	143.6	35.0	1598
0.7	52.0	3078	132.3	32.3	2238
1	35.0	2959	127.2	31.0	3197
2	16.5	2790	120.0	29.3	6395
3	9.8	2486	106.9	26.1	9593
4	6.0	2029	87.2	21.3	12790
5	3.5	1479	63.6	15.5	15988
TiNO thin film sample					
0.5	19.9	871	36.0	9.1	1656
0.7	11.0	674	28.0	7.0	2319
1	7.5	657	27.2	6.9	3313
2	3.0	525	21.8	5.5	6626
3	1.9	499	20.7	5.2	9939
4	1.4	490	20.3	5.1	13253
5	1.0	473	19.6	4.9	16566
